# Hyaloid vasculature and mmp2 activity play a role during optic fissure fusion in zebrafish

**DOI:** 10.1038/s41598-020-66451-6

**Published:** 2020-06-23

**Authors:** Megan L. Weaver, Warlen P. Piedade, Nishita N. Meshram, Jakub K. Famulski

**Affiliations:** Department of Biology, University of Kentucky, Lexington, KY USA

**Keywords:** Disease model, Biological metamorphosis

## Abstract

Vertebrate retinal development requires timely and precise fusion of the optic fissure (OF). Failure of this event leads to congenital vision impairment in the form of coloboma. Recent studies have suggested hyaloid vasculature to be involved in OF fusion. In order to examine this link, we analyzed OF fusion and hyaloid vasculogenesis in the zebrafish pax2a noi mutant line. We first determined that pax2a^−/−^ embryos fail to accumulate F-actin in the OF prior to basement membrane (BM) degradation. Furthermore, using 3D and live imaging we observed reduced OF hyaloid vascularization in pax2a^−/−^ embryos. When examining the connection between pax2a loss of function and hyaloid vasculature, we observed significant reduction of *talin1* expression, a regulator of hyaloid vasculature. In addition, cranial VEGF expression was found to be reduced in pax2a^−/−^ embryos. Pharmacological inhibition of VEGF signaling phenocopied the pax2a^−/−^ vasculature, F-actin and BM degradation phenotypes. Lastly, we determined that OF associated hyaloid vasculature is a source of *mmp2, mmp*14*a and mmp14b* expression and showed that mmp2 is functionally necessary for degradation of OF BM. Taken together we propose a pax2a driven mechanism that ensures proper and timely hyaloid vasculature invasion of the OF in order to facilitate availability of the BM remodeler mmp2.

## Introduction

Ocular development is a highly conserved process amongst vertebrate species. Assembly of the hemispherical, retinal structure from an initially flat sheet of cells requires many complex morphogenetic movements. One such morphogenetic movement involves the invagination of the optic vesicle which results in a fissure forming at the ventral region of the developing retina. This fissure, known as the choroid or optic fissure (OF), enables hyaloid vasculature cell migration into the developing retina and subsequent establishment of the hyaloid vasculature. Hyaloid vasculature is a temporary circulatory system required for ocular development, and in most cases will degenerate once mature blood vessels begin to grow^1–4^. As soon as the hyaloid vasculature has been established, the two opposing retinal epithelial sheets of the OF will undergo fusion. Thereby, they encase the ganglion cell axons localized in the optic stalk and complete retinal morphogenesis. Failure of OF fusion leads to a congenital blinding disorder known as coloboma^5–7^. Coloboma is a prevalent cause of pediatric blindness, accounting for approximately 10% of cases worldwide^6,8^. This makes it one of the leading causes of pediatric blindness. Coloboma is a spectrum disorder presenting unilaterally or bilaterally and ranging in severity from minor visual impairment, to complete blindness in the affected eye^9^. This spectrum of severity is associated with the location and degree to which the OF was able to fuse and the severity of subsequent loss of ganglion cell axons^7^.

Coloboma has been studied for many decades in many different species. Work over this time has led to a general outline of the signaling and morphogenetic pathways required for proper OF formation and fusion (recently reviewed in^10^). In particular, opposing action of bone morphogenetic protein (BMP) and sonic hedgehog (Shh) signaling establishing the dorsal-ventral pattern of the optic vesicle and ensuring proper expression of optic stalk and OF regulators pax2, vax1 and vax2^11,12^. However, the actual molecular mechanisms driving OF fusion remains largely unknown. The process of epithelial tissue fusion is not unique to the eye and occurs throughout development, encompassing neural tube closure, palatal shelf formation and eyelid development^13^. Epithelial fusion has been studied for over a century, and is known to involve transcriptional regulation, cell signaling pathways and morphogen gradients^14,15^. The actin cytoskeleton is thought to be a crucial component of the machinery driving epithelial fusion in many tissues^16^. The importance of the actin cytoskeleton during epithelial fusion involves lamellipodial and filopodial projections between the two opposing epithelia. These help to “zipper” the cells together to form a single continuous sheet. When lamellipodial and filopodial projections are precluded, epithelial fusion often fails^17^. Lamellipodia and filopodia have been observed during OF fusion almost 3 decades ago^18–20^. However, the functional and regulatory mechanisms behind these projections remain unknown. Another cellular mechanism known to be directly involved in epithelial fusion is the degradation of the basement membrane (BM). During epithelial fusion, the BM acts as a physical barrier restricting the establishment of cell-cell contacts, which must be removed in order to complete fusion. Recent work from several labs, working on different species, has characterized progressive removal of the BM during OF fusion^21–24^. However, the molecular mechanisms facilitating this process, in particular BM degradation, also remain largely unknown. It was recently suggested that hyaloid vasculature cells migrating through the OF could potentially signal or facilitate BM degradation^22^. Hyaloid vasculature nourishes the developing retina and lens while connecting to the choroid vasculature for proper blood flow^3^. Hyaloid vasculogenesis takes advantage of an open OF so that vasculature cells can migrate into the developing optic cup. Once OF fusion is completed, hyaloid vasculature is fully established. James *et al*. 2016, showed that mutations in zebrafish *talin1* (*tln1*), an actin cytoskeleton scaffolding protein known to be required for endothelial cell migration^25^, result in OF fusion defects. Theirs, and previous studies also indicated that *cloche* mutants, which lack all early hyaloid vasculature, have delayed BM breakdown in the region of the OF^22,26^. Since the hyaloid vasculature requires an open fissure to complete establishment of its network, it has been proposed that migrating hyaloid vasculature cells may regulate the timing of fissure fusion. This mechanism could potentially involve vasculature-mediated activation of the fusion machinery within the retinal rim cells, or direct supply of molecular factors, such as matrix proteases^27^. In support of this hypothesis, recent optic cup transplantation experiments in zebrafish embryos confirm that in the absence of hyaloid vasculature, ectopic retinal OFs fail to initiate fusion^28^. Hence, there is a clear link between OF fusion and hyaloid vasculature.

In our current study, we have undertaken a detailed analysis of zebrafish OF fusion in the pax2a^−/−^ coloboma model^29^. This included characterizing the timing of BM degradation, cytoskeletal responses, morphological apposition and hyaloid vascularization. When comparing pax2a^+/+^ and pax2a^−/−^ eyes we discovered decreased OF hyaloid vascularization and OF fusion failure. In particular we found that pax2a^−/−^ embryos exhibit a decrease in *tln1*, hyaloid vasculature makers as well as *VEGFaa, VEGFab* and VEGF*c* expression. Modulation of vascularization via pharmacological inhibition of VEGF signaling phenocopied the pax2a^−/−^ hyaloid vasculature and coloboma phenotypes. Mechanistically, we also show that hyaloid vasculature is a source of *mmp2*, *mmp14a* and *mmp14b* and that mmp2 activity is necessary for OF BM degradation. Taken together, we propose a novel pathway for the regulation of OF fusion where pax2a mediates proper timing and abundance of hyaloid vasculature cell recruitment to the OF and subsequent vasculature supplied mmp2-dependent BM degradation.

## Results

### Optic fissure basement membrane degradation is preceded by F-actin accumulation

**Figure 1:**
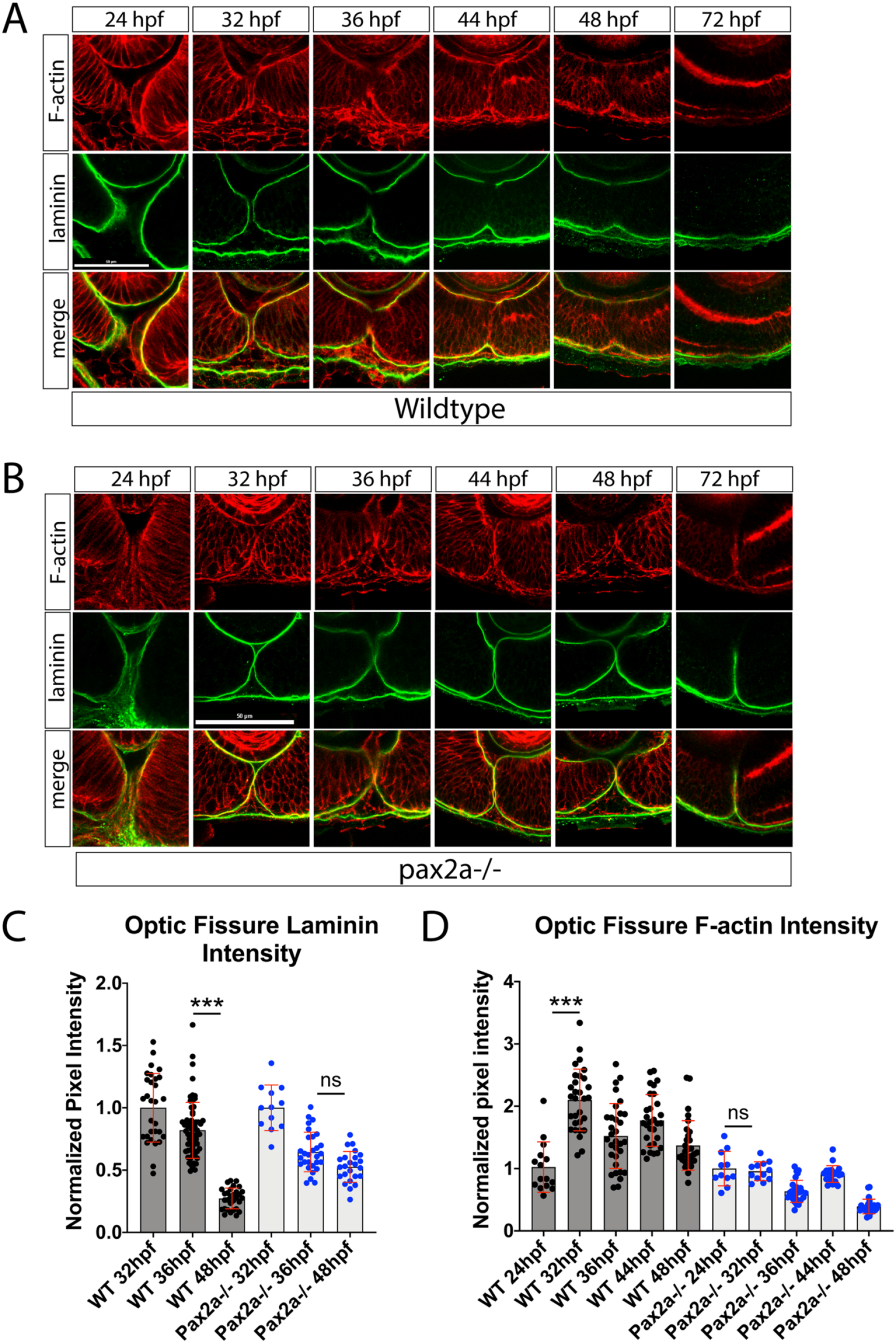
An increase in F-actin dynamics preceding laminin degradation during optic fissure fusion is disrupted in pax2a^−/−^ embryos. (**A**) Whole mount immunohistochemistry was used to simultaneously visualize F-actin (red) and laminin (green) during OF fusion, 24-72 hpf. Central-proximal sections obtained using confocal imaging were collected and quantified. Scale bar = 50 μm. (**B)** Whole mount Immunohistochemistry was used to simultaneously visualize F-actin (red) and laminin (green) during OF fusion, 24-72 hpf, in pax2^−/−^ embryos. Central-proximal regions of the OF are displayed. Scale bar = 50 μm. (**C)** Quantification of laminin signal intensity within the OF, normalized to regions of laminin staining juxtaposed to the lens. Relative pixel intensities are displayed. ANOVA p < 0.0001. (**D)** Quantification of F-actin intensity (phalloidin staining) within the OF, normalized to regions of F-actin signal within the lobe of the retina. Relative pixel intensities are displayed. ANOVA p < 0.0001.

Several recent studies have undertaken a detailed time course to map out the exact timing of OF fusion in numerous species, including zebrafish^21–24^. Overall, in zebrafish, the data point to ~32-36 hpf as the time of OF fusion initiation, as observed by BM degradation. To decipher the molecular mechanisms regulating OF fusion we also performed a detailed time course analysis of OF BM degradation using laminin immunohistochemistry (IHC) while additionally analyzing F-actin. Our goal was to determine whether changes in F-actin levels in the OF were correlative with fusion. James *et al*., 2016 had recently suggested that infiltrating vasculature endothelial cells migrating through the fissure could be a source of signal for fissure fusion. In fact, they had shown that interactions between vasculature and the fissure resulted in an increase of F-actin^22^. As such, we first performed whole mount IHC for laminin deposition in embryos starting at 24 hpf. We sampled every 4 hours up to 48 hpf and then at 56 and 72 hpf (Fig. 1A). To analyze the progression of the fusion process, we quantified the laminin signal within the OF from 3D confocal scans. We quantified distal, medial and proximal regions of the fissure (Fig. S1A). Regions of the BM outside the OF and juxtaposed to the developing lens were used to normalize the laminin signal (Fig. S1B). When comparing results from the three regions we did not observe significant differences in the distribution of laminin or F-actin (Fig. S1C). Going forward we focused on quantifying laminin signal in the central proximal region (medial) of the fissure (Fig. 1C). This region has been previously shown to represent the site of fusion initiation, is known to interact with hyaloid vasculature and is easily identified in 24-72 hpf embryos^22^. In agreement with recent studies in zebrafish, we found that OF fusion initiates at ~32-36 hpf and does so in a central region of the fissure, subsequently proceeding proximally and distally. From observing distance between the fusing lobes of the retina in our time course for OF laminin status, Fig. 1A, we observed apposition to complete between 36-44 hpf (Fig. 1A). By 48 hpf, we observed most of the laminin signal was removed from the OF, and by 72 hpf, little to no laminin persists in the region (Fig. 1A,C).

To track F-actin levels during fusion we stained the laminin labeled embryos with phalloidin and quantified the signal within the fissure. To normalize phalloidin staining intensity we used an interior region of the retina (Fig. S1B). Our analysis revealed an increase in OF associated F-actin signal between 24-32 hpf, preceding the time that BM degradation is initiated (Fig. 1D). The observed increase of F-actin signal correlates with the timing of hyaloid vasculature migration(24-32 hpf) into the fissure. The nature of our assay did not precisely identify the cellular source of F-actin but it likely predominantly consisted of retinal rim and hyaloid vasculature cells. Taken together, we conclude that BM degradation is preceded by an increase of F-actin within the OF.

### Optic fissure fusion mechanics are disrupted upon loss of pax2a function

In order to test our hypothesis that an increase in F-actin is involved in the initiation of OF fusion we compared our findings to an established model of coloboma, the pax2a noi line^29^. Pax2 is required for OF fusion in several model systems and has been documented in human coloboma cases^30–32^. The noi mutation is predicted to result in a loss of pax2a function due to a premature stop codon at position 198^33^. Originally characterized for their no-isthmus phenotype, pax2a^n^°^i/n^°^i^ embryos (referred to as pax2a^−/−^ from this point on) elicit a fully penetrant unfused OF^34,35^. Unfortunately, homozygous mutants are not viable and do not enable the study of coloboma at juvenile or adult stages. We hypothesized that examining the molecular events leading to OF fusion in the pax2a noi system would inform us whether these events are functionally important. Similar to our WT study, we examined apposition, laminin and F-actin levels throughout the time of OF fusion (Figs. 1B,D, S2). In stark contrast to WT, pax2a^−/−^ embryos did not exhibit a significant decrease in laminin signal between 32-48 hpf. In fact, as expected, laminin appeared to be largely retained in the OF of pax2a^−/−^ embryos up to 72 hpf (Fig. 1B,C). Previous studies in pax2a null mice had also indicated failure of BM degradation and retention of laminin^32^. While there is a moderate decrease in laminin signal in pax2a^−/−^ embryos from 32-36 hpf (p = >0.0001), there was no significant loss of laminin signal between 36-48 hpf. This result is significantly different in WT embryos (p = >0.0001) which display a major decrease in laminin signal during this time. IHC images clearly show that laminin persists in the pax2a^−/−^ fissure up to and including 72 hpf (Fig. 1B). When examining OF F-actin levels in pax2a^−/−^ embryos we did not detect the expected increase between 24 and 32 hpf (Fig. 1D). In fact, up to 48 hpf F-actin levels measure significantly lower than in WT embryos. Similar results were also observed when quantifying distal and proximal regions of the fissure (Fig. S1D). Our data therefore suggest that the absence of OF F-actin accumulation between 24 and 32 hpf, correlates with failure to initiate fusion. Lastly, when examining retinal lobe distance in pax2a^−/−^ embryos we did not observe any significant defects in the degree of apposition up to 48 hpf (Fig. S2). Taken together, we conclude that in the absence of pax2a, F-actin fails to accumulate in the OF and this correlates with the failure to degrade the BM. Based on our findings, we therefore conclude that retinal lobes still become apposed by 48 hpf in pax2a mutants. In summary, we propose that accumulation of F-actin is necessary for the initiation of OF BM degradation. We therefore next sought to investigate the source of OF F-actin accumulation.

### Loss of pax2a function leads to reduced hyaloid vasculature within the optic fissure

**Figure 2:**
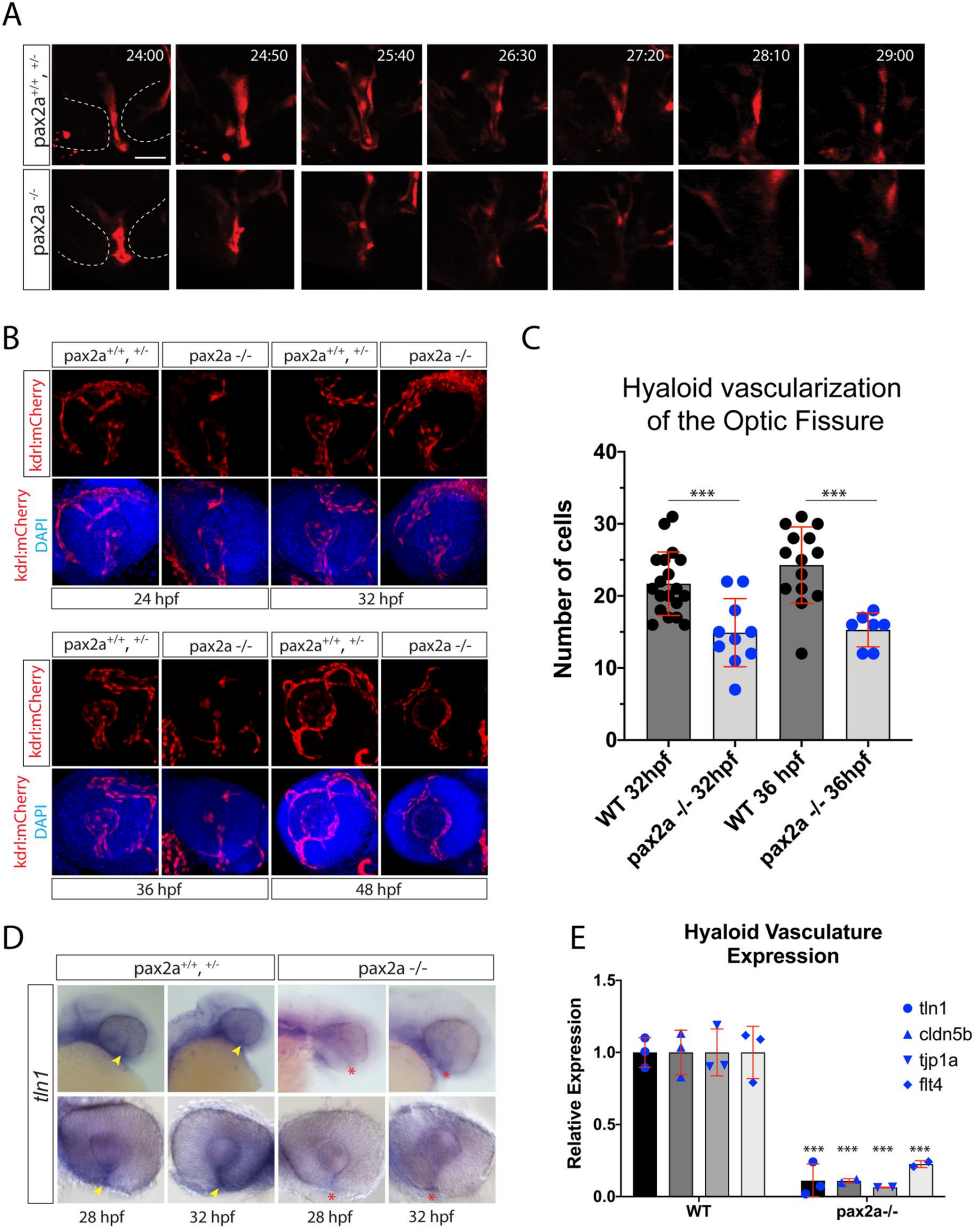
Pax2a is necessary for recruitment of hyaloid vasculature into the optic fissure. (**A)** *In vivo* 4D confocal imaging of Tg[*kdrl*:mCherry] pax2a^+/+,+/−^ or pax2a^−/−^ embryos. Time lapse series depicting the region of the OF (dotted white lines) and mCherry positive vasculature endothelial cells migrating through the fissure from 24-29 hpf. Scale bar = 10 μm. (**B)** Comparison of pax2a^+/+,+/−^ and pax2a^−/−^ vascularization during OF fusion, 24-48 hpf. 3D reconstructions of whole mount Tg[*kdrl*:mCherry] (red) pax2a^+/+,+/−^ or pax2a^−/−^ embryos with DAPI (blue) stained DNA. Scale bar = 50 μm. (**C)** Quantification of the number of mCherry positive cells from 3D confocal stacks within the region of the OF. Individual embryo results are depicted. ANOVA p < 0.0001. (**D)** Whole mount *in situ* hybridization comparing *tln1* expression between pax2a^+/+,+/−^ and pax2a^−/−^ embryos at 28 and 32 hpf. *tln1* signal within the OF (yellow arrowhead) appears reduced in Pax2a^−/−^ embryos (red *). (**E)** qPCR analysis of *tln1* expression from heads of pax2a^+/+^ and pax2a^−/−^ embryos at 32 hpf confirms a reduction in *tln1* expression.

Work by James *et al*. 2016, suggested that F-actin accumulation in the OF may result from interaction between retinal rim cells and invading hyaloid vasculature. To test this possibility, we examined hyaloid vasculature in pax2a^−/−^ Tg[kdrl:mCherry] embryos. Using 3D *in vivo* time-lapse confocal microscopy we recorded migration of mCherry expressing cells through the OF from 24 to 30 hpf (Fig. 2A). At 24 hpf, both WT and pax2a^−/−^ embryos contain mCherry expressing cells within the fissure. However, over the next six hours of imaging it is apparent that pax2a^−/−^ embryos have significantly fewer mCherry expressing cells pass through the OF (Fig. 2A, Movie 1,2). To visualize this effect, we fixed WT and pax2a^−/−^ Tg[kdrl:mCherry] embryos at 24, 32, 36 and 48 hpf to collect and render 3D confocal stacks (Fig. 2B). Starting as early as 32 hpf, we noticed a clear reduction in the number of vasculature cells within the OF and retina. Furthermore, we counted the number of mCherry positive cells found within the OF at 32 and 36 hpf (Fig. 2C). The data indicated that in pax2a^−/−^ embryos there is a significant reduction in the number of mCherry expressing cells at both 32 and 36 hpf (Fig. 2C). Additionally, using 3D rendering, we noted that in 48 hpf pax2a^−/−^ embryos the hyaloid vasculature established in the back of the lens is reduced in size and lacks proper connections to the newly forming choroidal and superficial vasculature systems (Movie 3,4). In the previously aforementioned study, James *et al*. 2016 also showed that tln1 is necessary for hyaloid vasculature recruitment to the OF and subsequent fusion. Since pax2a^−/−^ embryos lack BM degradation, fail to initiate F-actin accumulation and have reduced hyaloid vascularization (Figs. 1, 2), we sought to examine *tln1* expression in this model. To examine *tln1* expression status we performed whole mount *in situ* hybridization (WISH) for *tln1* comparing WT siblings to pax2a^−/−^ mutant embryos. WT expression of *tln1* was observed in the OF between 28 and 48 hpf coinciding with pax2a expression (Figs. 2D, S3). In pax2a^−/−^ embryos, OF *tln1* expression appears significantly reduced compared to WT while retaining similar expression in periocular regions (Fig. 2D). *Tln1* expression is also reduced in the mid brain-hind brain boundary, another region of strong *pax2a* expression (Figs. 2D, S3A). Our WISH data was further supported by qPCR results showing a significant decrease in *tln1* expression at 32 hpf (Fig. 2E). Finally, to verify our observations were indicative of reduced hyaloid vasculature we also analyzed expression of additional vasculature markers *flt4, tjp1a* and *cldn5b*^36,37^. qPCR comparison of 32 hpf WT and pax2a^−/−^ embryo heads indicated a significant decrease of *flt4*, *tjp1a* and *cldn5b* expression, validating our observation of overall reduced vasculature in mutant embryos (Fig. 2E). Overall, our data indicate that pax2a^−/−^ embryos exhibit decreased expression of *tln1* and impaired hyaloid vascularization of the OF.

### Inhibition of VEGF signaling impairs optic fissure fusion mechanics

**Figure 3:**
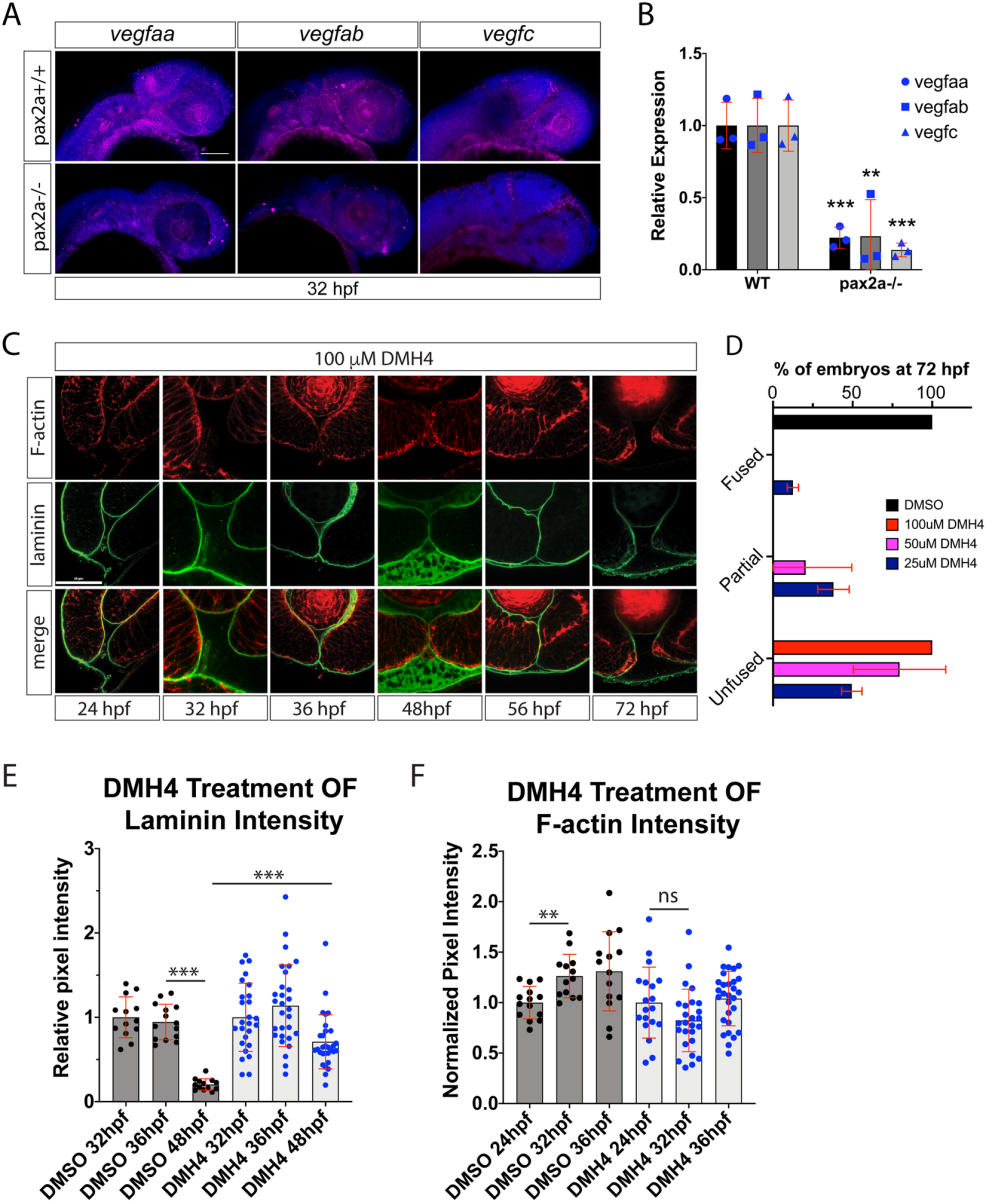
Inhibiting angiogenesis disrupts optic fissure fusion mechanics. (**A)** Fluorescent whole mount *in situ* hybridization of *vegfaa, vegfab* and *vegfc* at 32 hpf in WT and pax2a^−/−^ embryos. Loss of pax2a results in decreased *vegfaa, ab* and *c* expression. DAPI is depicted in blue. Scale bar = 100 μm. (**B)** qPCR results for *vegfaa, vegfab* and *vegfc* expression from heads of pax2a^+/+^ and pax2a^−/−^ embryos at 32 hpf. (**C)** 3D confocal images of Tg[*kdrl*:mCherry] (red) embryos treated with 100 μM DMH4 between 24-72 hpf. DNA was stained with DAPI (blue). Scale bar = 50 μm. (**D)** Quantification of 48 hpf DMH4 treated embryos for fissure fusion (absence of laminin signal), partial fusion (partial retention of laminin) or failure to fuse (retention of laminin throughout the fissure). n = 22 (DMSO), 52 (100 μM), 22 (50 μM), 24 (25 μM) ANOVA p < 0.0001. (**E)** Quantification of laminin signal intensity within the central-proximal region of the OF, normalized to regions of laminin staining juxtaposed to the lens. Relative pixel intensities are displayed. ANOVA p < 0.0001. (**F)** Quantification of F-actin signal intensity (phalloidin staining) within the central-proximal region of the OF, normalized to regions of F-actin signal within the lobe of the retina. Relative pixel intensities are displayed. ANOVA p < 0.0001.

Based on our discovery of impaired OF hyaloid vasculature in pax2a^−/−^ embryos, we next examined whether this phenomenon is associated with failure of OF fusion. Hence, we turned our attention to vascular endothelial growth factor (VEGF) signaling. VEGF, the ligand for vascular endothelial growth factor receptor (VEGFR), is a prime candidate for regulating the migration and proliferation of hyaloid vasculature cells. In support of this notion, when examining expression patterns for VEGF ligands, vegfaa, ab, and c we found *vegfaa, ab*, and *c* to be expressed in the head and periocular regions (Fig. 3A). In order to test for a link between the loss of pax2a function and VEGF signaling we analyzed *vegfaa*, *ab* and *c* expression in pax2a^−/−^ embryos. Fluorescent wholemount *in situ* hybridization (FWISH) at 32 hpf indicated a decrease of *vegfaa*, *ab* and *c* expression in the cranial regions of mutant embryos (Fig. 3A). These results were also supported by qPCR (Fig. 3B). Our data therefore suggest that a decrease in VEGF signaling may be a factor responsible for reduced hyaloid vascularization of the OF in pax2^−/−^ embryos. This finding may also explain the severe vascular phenotypes pax2a^−/−^ embryos develop, in particular heart edema and other cardiac misfunction.

In order to test whether VEGF signaling plays a direct role in hyaloid vascularization of the OF and subsequently fissure fusion we sought to inhibit VEGF activity. We therefore took advantage of a dorsomorphin derivative, DMH4, which has been shown in zebrafish to selectively inhibit VEGF signaling independent from BMP^38^. Based on published working concentrations, we conducted a dose response to examine DMH4 effects on hyaloid vasculature using Tg[*kdrl*:mCherry] as a readout^38^. Treatment of embryos from 12-24 hpf, ranging from 1-100 μM, resulted in a dose dependent reduction of mCherry signal in the developing retina (Fig. S4A). We decided to use the 100 μM concentration for subsequent experiments as this concentration was able to completely inhibit vascularization of the retina up to 56 hpf without any significant impact on overall embryo health (Fig. S4B,C). Embryos were treated starting at 12 hpf and examined for fissure fusion status via whole mount laminin IHC at 24, 32, 36, 48, 56 and 72 hpf (Fig. 3C). 3D confocal imaging revealed a persistence of laminin signal within the fissure up and including 72 hpf in all embryos examined (Fig. 3C). This was in contrast to DMSO treated embryos which exhibited no defects in OF fusion (Fig. S14D). Long term treatment with 100 μM DMH4, 56 hpf + , did result in a degree of retinal toxicity (Fig. S4B). To circumvent this effect, we also treated embryos from 12-72 hpf with 25 and 50 μM DMH4 (Fig. S4B). Toxicity appeared reduced at the lowest concentration, 25 μM, while still eliciting a failure of OF fusion phenotype in 87.5% of treated embryos (Figs. 3D, S4E). Observed retention of OF laminin signal at 48 hpf in DMH4 vs DMSO treated embryos generated an overall pattern of results very similar to what we observed in pax2a^−/−^ (Fig. 3E). While DMH4 treatment did result in a significant reduction of OF laminin signal from 36-48 hpf (p = <0.0002), OF laminin levels of DMH4 treated embryos were significantly higher than DMSO controls at 48 hpf (Fig. 3E). When measuring the degree of retinal lobe apposition at 48 hpf, we saw no negative effects of DMH4 treatment. This suggests that the observed failure of OF BM degradation is unlikely to result from optic cup morphogenesis delay (Fig. S2). However, it remains possible that apposition is initially delayed by DMH4 treatment yet completes by 48 hpf. In addition to laminin, we also imaged and quantified OF F-actin levels during DMH4 treatment (Fig. 3F). Similar to pax2a^−/−^ embryos, DMH4 treatment prevented the accumulation of OF F-actin between 24-32 hpf (Fig. 3F). This was in contrast to DMSO treated embryos which exhibited the expected accumulation of F-actin in the fissure between 24-32 hpf.

**Figure 4:**
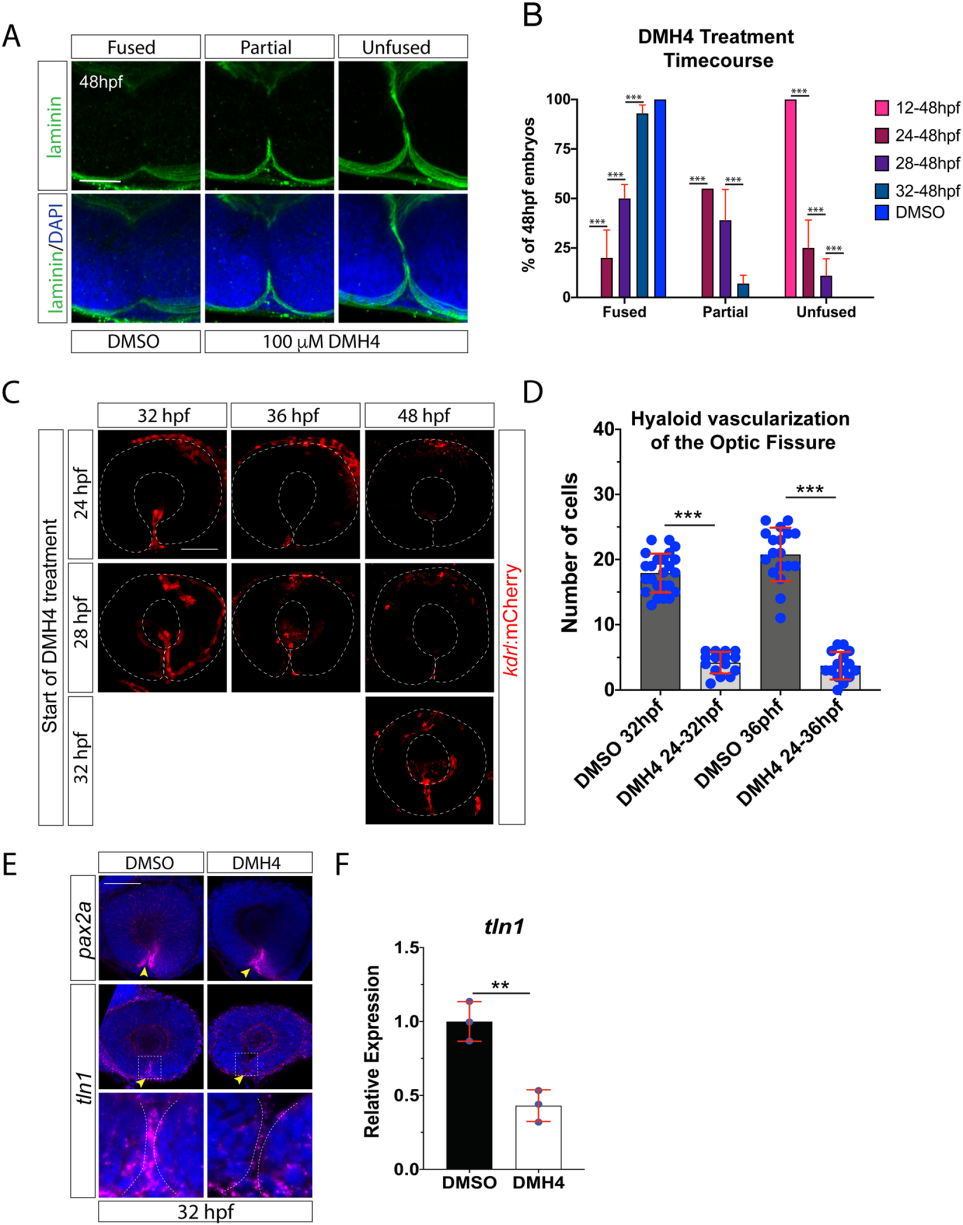
VEGF signaling is plays a role in optic fissure fusion. (**A)** Whole mount Immunohistochemistry was used to visualize laminin (green) at 48 hpf after DMSO or DMH4 treatment. Central-proximal regions of the OF are displayed depicting fused, partially fused or unfused optic fissures. Scale bar = 50 μm. (**B)** Quantification of 48 hpf DMH4 treated embryos for fissure fusion (absence of laminin signal), partial fusion (partial retention of laminin) or failure to fuse (retention of laminin throughout the fissure). n = 41(12-48 hpf), 38(24-48 hpf), 38(28-48 hpf), 44(32-48 hpf), 40 (DMSO). ANOVA p < 0.0001. (**C)** 3D reconstructions of whole mount Tg[*kdrl*:mCherry] embryos treated with 100 μM DMH4 at various time points. Broken white line outlines the retina. Scale bar = 50 μm. (**D)** Quantification of the number of mCherry positive cells from 3D confocal stacks within the region of the OF after DMSO or DMH4 treatment. Individual embryo results are depicted. ANOVA p < 0.0001. (**E)** Fluorescent whole mount *in situ* hybridization of *pax2a* or *tln1* probe at 32 hpf in DMSO or DMH4 treated embryos. OF expression is indicated with a yellow arrowhead. DMH4 treatment does not appear to alter *pax2a* expression but eliminates *tln1* expression from the OF. Broken white lines outline the retinal lobes. Scale bar = 50 μm (**F)** qPCR results for *tln1* expression in DMH4 treated embryos.

To show timing specificity of our DMH4 treatments we also performed a time course analysis. When treating embryos with DMH4 starting at 12 hpf we observed total inhibition of fissure fusion, indicated by retention of OF laminin signal at 48 hpf. However, when DMH4 was added at later time points, 24, 28 or 32 hpf we observed a corresponding decrease in fissure fusion failure (Fig. 4A,B). Interestingly, starting DMH4 treatment at 32 hpf had little effect on OF fusion. To correlate our DMH4 time course treatment to hyaloid vasculature, we imaged vasculature using the Tg[*kdrl*:mCherry] line (Fig. 4C). Starting DMH4 treatment at 24 or 28 hpf significantly reduced the number of hyaloid vasculature cells in the OF at both 32 and 36 hpf and by 48 hpf, little to no vasculature was observed (Fig. 4C). Quantification of hyaloid cells within the fissure at 32 and 36 hpf supported these observations (Fig. 4D). Importantly, the degree of OF hyaloid vascularization correlated with the rates of OF fusion failure observed (Fig. 4B). Significant reduction directly correlated to an increase in fusion failure (Fig. 4B). On the other hand, delay of DMH4 treatment until 32 hpf did not eliminate all hyaloid vasculature in the OF at 48 hpf and correlated with increased rates of OF fusion (Fig. 4B). Sufficient hyaloid vasculature recruitment to the OF is therefore critical at early stages of OF fusion, 24-28 hpf.

Lastly, in order to confirm the source of tln1 in the OF as hyaloid vasculature, we assessed *tln1* expression after DMH4 treatment. Fluorescent whole mount *in situ* hybridization (FWISH) indicated a significant decrease of *tln1* but not *pax2a* expression in DMH4 treated embryos (Fig. 4E). qPCR analysis supported our observation (Fig. 4F). This suggests that *tln1* is most likely expressed by the vasculature cells within the OF. Based on these findings, we conclude that inhibition of VEGF signaling results in failure of F-actin accumulation and basement membrane breakdown due to the absence of hyaloid vasculature cells in the OF.

### Hyaloid vasculature is a source of mmp2 necessary for OF BM degradation

**Figure 5:**
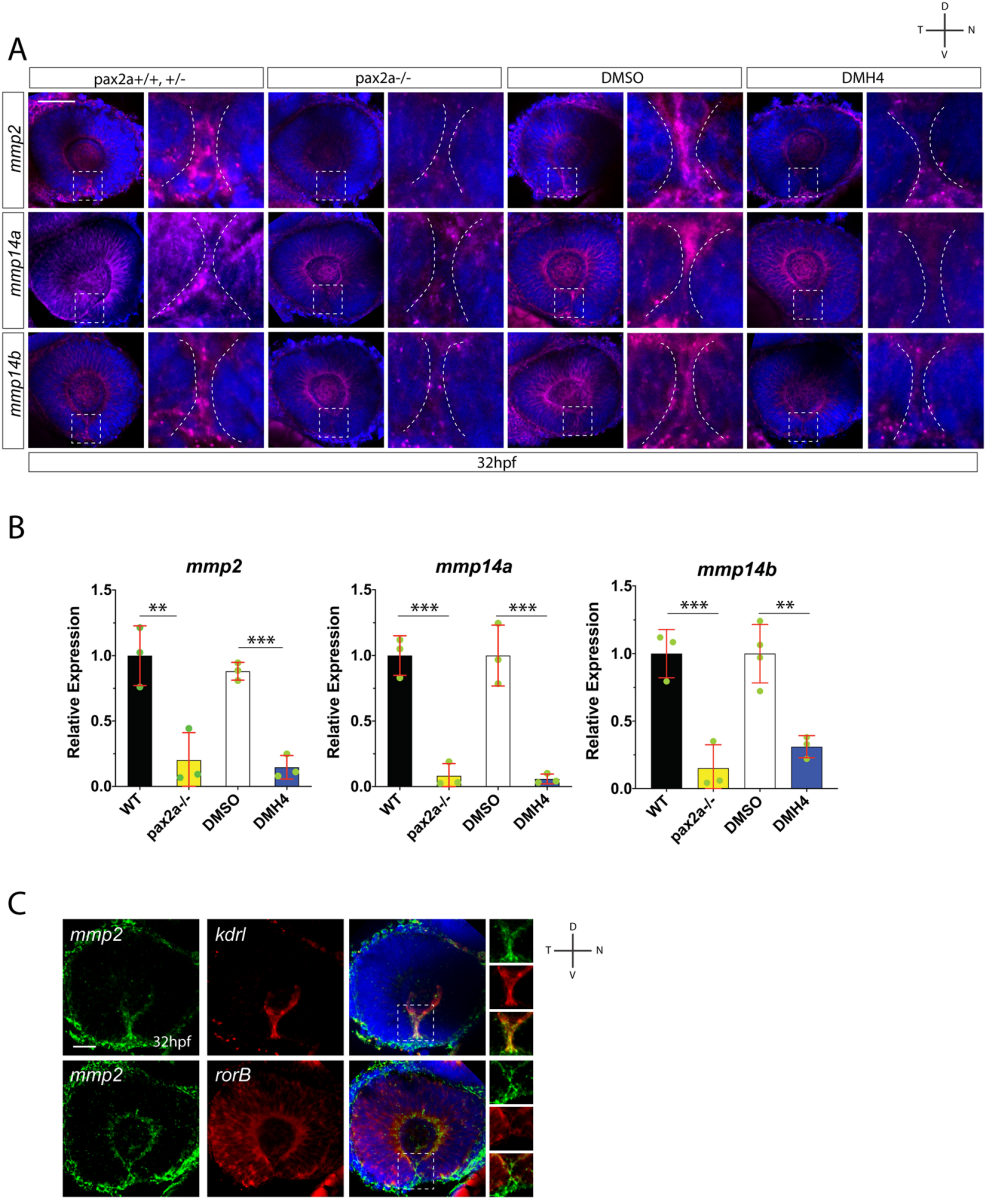
Hyaloid vasculature is a source of mmp2 during optic fissure fusion. (**A)** Fluorescent whole mount *in situ* hybridization comparing *mmp2, mmp14a* and *mmp14b* expression in WT vs pax2a^−/−^ and DMSO vs DMH4 treated embryos at 32 hpf. Broken white lines outline the OF retinal lobes. *mmp2, 14a* and *14b* expression within the OF is reduced in pax2a^−/−^ and DMH4 treated embryos. Scale bar = 50 μm. (**B)** qPCR analysis confirms a decrease in expression of *mmp2, 14a* and *14b* in both pax2a^−/−^ and DMH4 treated embryos. (**C)** Two color whole mount *in situ* hybridization simultaneously examining *mmp2* and *kdrl*, or *mmp2* and *rorB* expression at 32 hpf. DNA was stained with DAPI. Scale bar = 50 μm. Clear overlap of signal is observed for *mmp2* and *kdrl*, but not *mmp2* and *rorB*.

The above data confirm and support a model where hyaloid vasculature drives or initiates the OF fusion process. However, a missing key to this model is the mechanism by which vasculature cells induce fissure fusion. James *et al*. 2016, along with others, have suggested vasculature cells may be a source of BM degradation enzymes, such as matrix metalloproteases (mmp). To investigate this further, we used WISH to examine mmp expression within the OF between 24-48 hpf. Our examination of mmp expression indicated that *mmp2, 14a* and *14b* were expressed within the fissure between 28-36 hpf (Fig. S5A and Famulski lab unpublished data). Mmp2 had recently been associated with OF fusion in the mouse while evidence from an mmp2 metalloproteinase activity probe in zebrafish indicated mmp2 activity is present in the developing eye and likely OF^39,40^. mmp14 is an activator of mmp2 and its co-expression with mmp2 within the fissure suggests mmp2 is in fact active^41,42^. To test whether mmp2 fits within our model we assayed *mmp2, mmp14a and mmp14b* expression in pax2a^−/−^ and DMH4 treated embryos. In both cases, OF expression of *mmp2, mmp14a and mmp14b* was reduced as observed by FWISH and confirmed by qPCR (Figs. 5A,B, S5B). This suggested that all three may be expressed by the hyaloid vasculature cells. To assess this, we performed two color FWISH for *mmp2* and *kdrl*. Confocal imaging verified that *mmp2* expression is co-localized with that of *kdrl* within the OF (Fig. 5C). Conversely, when performing two color FWISH for *mmp2* and *rorB*, a retina specific probe, we did not detect co-localization of the signals. We therefore conclude that the source of mmp2 in the OF is most likely hyaloid vasculature cells.

**Figure 6:**
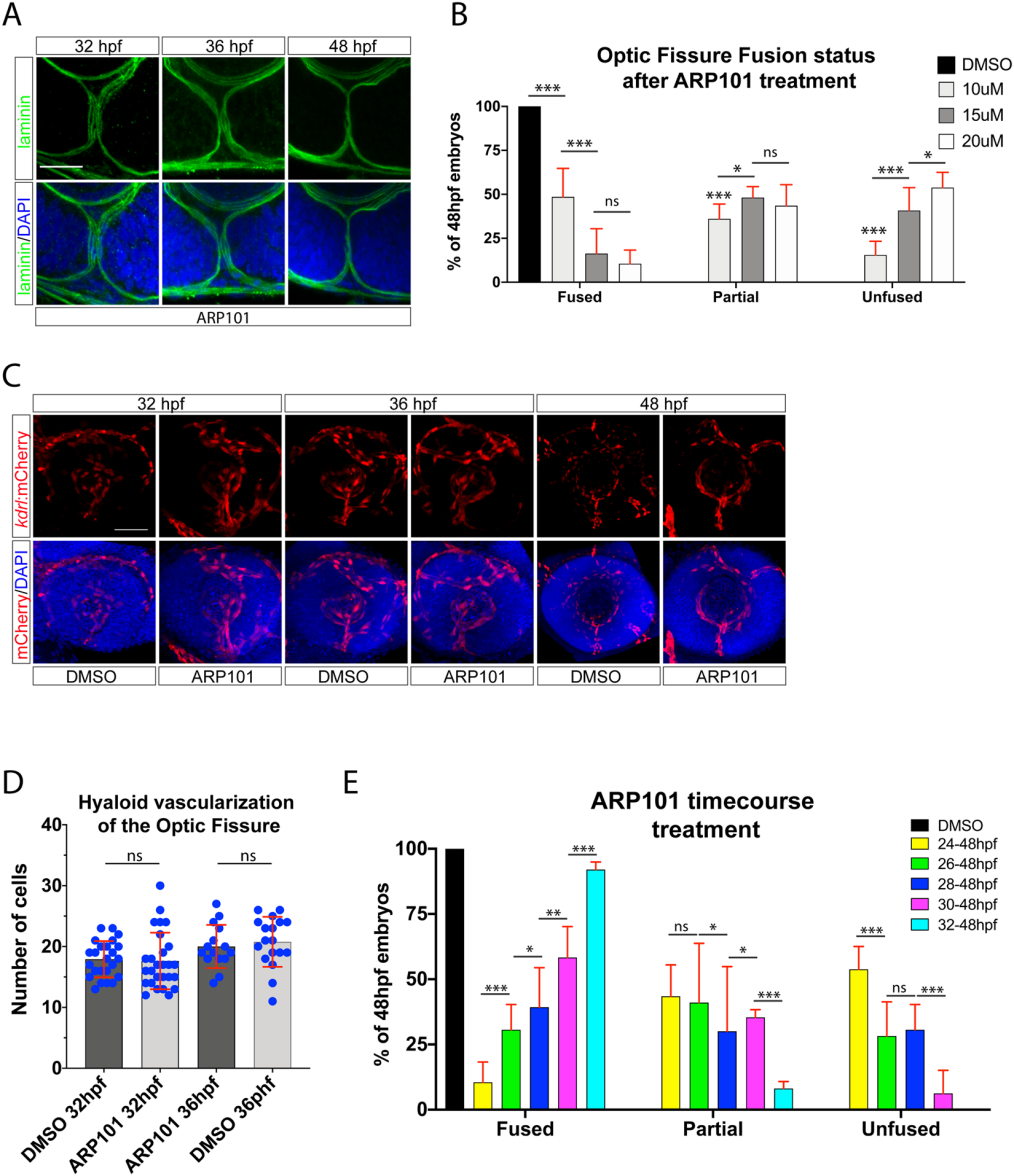
Proper timing of mmp2 activity is required for optic fissure fusion. (**A)** Whole mount Immunohistochemistry was used to visualize laminin (green) and DAPI (blue) in ARP101 treated Tg[*rx3*:GFP] embryos at 32, 36 and 48 hpf. Stacks of central-proximal regions of the OF are displayed. Scale bar = 50 μm. (**B)** Quantification of 48 hpf ARP101 treated embryos for fissure fusion (absence of laminin signal), partial fusion (partial retention of laminin) or failure to fuse (retention of laminin throughout the fissure). n = 41 (10 μM), 38 (15 μM), 42 (20 μM) ANOVA p < 0.0001. (**C)** 3D reconstructions of whole mount Tg[*kdrl*:mCherry] (red) embryos treated with 20 μM ARP101 from 24-32, 36 or 38 hpf. DNA was stained with DAPI (blue). Scale bar = 50 μm. (**D)** Quantification of the number of mCherry positive cells from 3D confocal stacks within the region of the OF after DMSO or 20 μM ARP101 treatment. Individual embryo results are depicted. (**E)** Quantification of 48 hpf ARP101 treated embryos for fissure fusion, partial fusion or failure to fuse after various treatment initiation times from 24-32 hpf. n = 42 (24-48 hpf), 29 (26-48 hpf), 29 (28-48 hpf), 23 (30-48 hpf), 37 (32-48 hpf). ANOVA p < 0.0001.

Finally, to determine whether mmp2 activity is necessary for OF fusion we treated embryos with ARP101, a specific mmp2 inhibitor previously shown to be effective in zebrafish^43^. Treating embryos with ARP101 from 24-48 hpf inhibited OF BM breakdown in a dose dependent manner (Figs. 6A, S6A,B). When compared to DMSO, embryos treated with 15 or 20 μM ARP101 either completely or partially retain their OF BM up to 48 hpf (Fig. 6B). To ensure that inhibition of mmp2 activity is not affecting hyaloid vasculature migration into the OF we imaged Tg[*kdrl*:mCherry] DMSO or ARP101 treated embryos from 24-48 hpf. OF associated vasculature appeared unchanged at 32, 36 and 48 hpf (Fig. 6C). Furthermore, quantification of hyaloid cells within the fissure at 32 and 36 hpf indicated that inhibition of mmp2 activity does not have any effects on OF vascularization (Fig. 6D). Lastly, we also examined *pax2a* expression in ARP101 treated embryos and did not observe any negative effects (Fig. S6C). We therefore concluded that mmp2 activity is necessary for BM breakdown in the OF. To determine when mmp2 activity is required we performed a time course of the ARP101 treatment starting between 24-32 hpf and assayed BM status at 48 hpf (Fig. 6E). Treatments started later than 30 hpf had little to no effect on OF BM degradation, indicating that mmp2 activity is optimally required between 24-30 hpf (Fig. 6E). This finding also correlates with the observed timing of *mmp2* expression in the OF (Fig. S5A). However, because we cannot determine exactly how quickly ARP101 inhibits enzymatic activity in our assay, active mmp2 may persist in the OF longer than 30 hpf. Lastly, in addition to pharmacological inhibition of mmp2 function, we also sought to examine the consequences of *mmp2* gene inactivation. To do so, we employed the recently introduced Alt-R-crRNA/Cas9 system which has been shown to provide highly effective CRISPR mediated F0 gene knockouts in zebrafish^44^. Injection of a pre-designed *mmp2* crRNA duplex with Cas9 enzyme resulted in ~53% of embryos displaying a coloboma like phenotype at 72 hpf (Fig. S7). Confocal imaging of laminin staining indicated a partial failure in OF BM degradation and persistence of a minor fissure. The observed phenotype was similar to but not as pronounced as observed in pax2^−/−^ embryos at 72 hpf. This may result from incomplete F0 phenotypes. As such, future examination of germline transmitted loss of function alleles of *mmp2* will be necessary to fully examine the functional consequence on OF fusion. Taken together, we propose that mmp2 activity is part of the mechanism involved in OF BM degradation and subsequent fusion of the fissure.

## Discussion

Studies of OF fusion dating back several decades have been suggesting a direct connection between the fusion process and hyaloid vasculature found within the fissure. In fact, this hypothesis has been recently strengthened by data showing that a reduction of hyaloid vasculature in the fissure, or removal of optic vesicles from sources of vasculature inhibits or significantly delays fusion^22,28^. In our study, we have characterized a pax2a driven mechanism that ensures proper vascularization of the OF and expression of BM remodelers *mmp2, 14a* and *14b*. In conclusion, our findings further validate the notion that hyaloid vasculature is an active and necessary component of the machinery driving OF fusion in zebrafish.

Several recent reports, including this one, have comprehensively characterized OF fusion timing^21–24^. Work from zebrafish, mice and chick all point to an orderly progression involving: **(1)** retinal growth and cellular rearrangement leading to nasal and temporal retinal lobe apposition, **(2)** invasion of the fissure by endothelial and neural crest cells forming the hyaloid vasculature system, **(3)** cellular signaling, either between retinal rim cells or between rim cells and the migrating vasculature cells, **(4)** degradation or removal of the basement membrane to enable physical connection of the rim cells and subsequent formation of a continuous retinal epithelial sheet via re-polarization and cell-cell adhesion. Step 1 has been nicely characterized in a few recent publications outlining the flow of retinal cells and morphological formation of the fissure^45–47^. For step 2, several reports have carefully characterized the formation of the hyaloid vasculature system, including migration of hyaloid vasculature precursor cells into the fissure as soon as it forms^1–3^. Importantly, perturbation of this process, or removal of the developing eye from its source, has been shown to lead to fissure fusion failure^22,28^. To date, steps 3 and 4 are the least understood. Recent work from our lab has characterized the composition of core BM components within the fissure^23^. Additionally, work from other labs has identified several molecular components associated with cell-cell adhesion and epithelial sheet fusion to function within the fissure, including β-catenin, n-cadherin, and netrin^22,24,48–51^. However, the timing and the molecular mechanism organizing and regulating these components remains uncharacterized. Finally, the elephant in the room has always been the identity of the BM degradation mechanism. To date, only adamts16 has been functionally examined in context of fissure fusion while mmp2 activity was detected during mouse OF fusion and mmp23bb was implicated in the fusion process from transcriptomic data^40,50,52^.

While attempting to address the mechanistic aspects of steps 3 and 4 using the pax2a noi model we first uncovered a relationship between F-actin and BM degradation. Work by *James et al. 2016* suggested that F-actin accumulation may be indicative of hyaloid cell interaction with retinal rim cells. During our detailed time course analysis, we discovered that BM degradation is in fact preceded by an increase in F-actin signal within the OF (Fig. 1). When assayed in pax2a^−/−^ embryos the F-actin accumulation is absent while the hyaloid vasculature in the OF is also diminished. The timing of F-actin accumulation coincides with the active migration of hyaloid vasculature cells through the fissure (Fig. 2). Furthermore, we showed pharmacologically, via VEGF inhibition, that hyaloid vasculature is necessary for the accumulation of F-actin and OF BM degradation. Transcriptionally, we showed that the decrease in hyaloid vasculature in pax2a^−/−^ embryos coincides with a significant decrease in *tln1* expression. Tln1 is a key regulator of endothelial cell migration, recently shown to be directly involved in OF fusion^22^. In fact, loss of *tln1* function has been previously shown to decrease OF associated vasculature and inhibit BM degradation^22^. In our study, we detected a significant decrease in *tln1* expression upon VEGF inhibition (DMH4 treatment), suggesting that *tln1* expression within the OF is associated with hyaloid vasculature. Tln1 is known to be a direct link between the actin cytoskeleton and the BM via integrin^53,54^, and has been associated with the formation of adhesion junctions^55,56^. One could therefore hypothesize several different models for its role in OF fusion. We predict that tln1 is regulating the ability of hyaloid vasculature cells to migrate properly through the OF, however we cannot rule out the possibility that tln1 directly participates in the OF fusion process. The absence of *tln1* expression is most likely a correlation to the decrease of hyaloid vasculature cells within the fissure of pax2a^−/−^ embryos. In support of this we observed a significant decrease in *tln1* expression upon DMH4 inhibition and therefore absence of hyaloid vasculature in the OF. As such, the decrease in *tln1* expression is likely indicative of decreased numbers of hyaloid vasculature cells which require *tln1* expression for proper migration into the fissure and or proliferation.

Our study strongly supports the notion that vasculature plays an integral part in OF fusion. Proper execution of angiogenesis is therefore a potential mechanism to ensure timely vascularization of the OF. The deficiency of hyaloid vasculature in pax2a mutant embryos may therefore result from a decrease in VEGF signaling. Using WISH and qPCR we detected a significant decrease in cranial *vegfaa*, *ab* and *c* expression. VEGF signaling is critical for angiogenesis and proper vasculogenesis throughout the embryo, a decrease in its activity is therefore likely to result in a series of deficiencies, including hyaloid vascularization of the OF. These findings supported the observation that pax2a mutants suffer from severe heart malfunction and subsequent edema and hemorrhage. The mechanisms of how pax2a regulates VEGF signaling remains unknown at this time and will need to be investigated in future studies. The observed decrease in VEGF expression does not directly correlate to regions of *pax2a* expression, which therefore suggests indirect regulation or negative VEGF feedback signaling during early development in the absence of pax2a function. In addition, phenotypes resulting from reduced VEGF signaling and therefore vasculogenesis may stem from subsequent circulation and cardiac function defects as well.

The last aspect of our proposed model pertains to the OF BM degradation mechanism. It had been suggested that vasculature may be the source of BM degradation activity during OF fusion^22^. To that end, we have discovered that *mmp2* is expressed within the hyaloid vasculature during OF fusion (Fig. 5). In fact, timing of *mmp2* expression correlates with the expected timing for initiation of BM degradation (Fig. S5). When we examine pax2a mutants or inhibit hyaloid vasculature completely (DMH4 treatment) we no longer detect expression of *mmp2* or *mmp14a* and *14b*. Delayed treatment with DMH4 also resulted in absence of *mmp2* expression (90% of embryos after 24-32 hpf treatment (n = 10) and 63% in 28-32 hpf treated embryos (n = 11). Furthermore, using ARP101, a specific mmp2 inhibitor, we showed that mmp2 activity is necessary for OF BM degradation, specifically between 26-32 hpf (Fig. 6). Mmp2 has been implicated in OF fusion previously. Mouse studies have shown *mmp2* expression within macrophages residing in the OF. Furthermore, recent examination of mmp2 activity, using a reporter construct, indicates that mmp2 is active in the eye and likely the OF^39^. Mmp14 is a known to activate mmp2 activity by cleaving the inhibitory pro-peptide of mmp2^41,42^. As such, co-expression of *mmp2* and *14* is further indicative of mmp2 playing a functional role in OF BM degradation. In the current study we did not directly assay the functional role of mmp14, but based previous work linking mmp2 activation to mmp14, it is likely that mmp14 is also going to be necessary for zebrafish OF fusion.

To date, the only BM degradation enzyme to be associated with OF fusion is adamts16. Loss of adamts16 function in zebrafish, via morpholino, led to a coloboma like phenotype which the authors credited to the inability to degrade laminin in the fissure^52^. We have not been able to reproduce *adamts16* expression within the fissure. Recent studies in mice, zebrafish and chick have compared expression in OF cells pre, during and post fusion^24,50,57,58^. Surprisingly, none of these studies identified any obvious candidates for carrying out BM degradation, including *mmp2* and *mmp14*. However, it remains possible that the OF tissue examined lacked the hyaloid vasculature and therefore prevented the identification of *mmp2* and *mmp14* as candidates. In addition, expression of *mmp2, 14a* and *14b* is transient in the fissure which may have contributed to their OF expression being missed by other groups. Our data indicate mmp2 activity is necessary for BM degradation of the OF, but it does not rule out a role for additional proteases. There may be several proteases involved in OF BM degradation, such as adamts16, mmp2 and others, yet inhibition of just one could trigger failure of the degradation process and lead to fissure fusion failure. Continuing studies into matrix protease activity will be needed to fully characterize the degradation mechanism(s) during OF fusion.

Recent studies of hyaloid vasculature during OF fissure fusion have also included examination of the cloche mutant line, which is considered to be avascular. Importantly, *cloche* mutants (clo^m39^) display a delay but not a complete lack of OF fusion^22^. This fact confounds our and other’s results that suggest hyaloid vasculature plays a role in OF fusion^28^. With our new finding that mmp2 plays a significant role in OF fusion we therefore examined *mmp2* expression in clo^m39^ mutants (Fig. S8). Interestingly, clo^m39^ embryos did display *mmp2* expression within the OF, albeit at visibly reduced levels. This finding suggests that clo^m39^ embryos either still retain a population of cells (possibly POM or NCC) that are found within the fissure and express *mmp2*, or that not all vasculature is eliminated in these mutants. On that note, previous examination of vasculature markers in clo^m39^ mutants indicated that *fli1* was still expressed in the retinal region suggesting some degree of vasculature may remain^59^. The persistence of even low levels of mmp2 may explain why OF fusion is delayed, but not eliminated in clo^m39^ mutant embryos. Furthermore, we also note that in our DMH4 treatments, which generate an avascular phenotype, we appear to block OF fusion completely, which is also in contrast to clo^m39^ mutants. This discrepancy may result from the absence of VEGF signaling which may be playing a secondary role to that of angiogenesis. An angiogenesis independent role of VEGF may involve signaling in other cell types such as the POM or NCC which are known to migrate through the OF. Future studies into mmp2 activity and VEGF signaling as well as interaction of POM and NCC with the OF will be of high priority.

In conclusion, we present a new model for the mechanism of OF BM degradation where pax2a functions to enable hyaloid vasculature invasion of the OF. Once in the fissure, vasculature cells express *mmp2, 14a* and *14b* to initiate BM degradation. As soon as fusion begins, vasculature becomes restricted from the fissure and ultimately the two retinal lobes fuse. Our model aligns with the molecular events observed in the fissure, including accumulation of F-actin at the time of vasculature migration through the fissure(24-28 hpf), expression of mmps(26-36 hpf) and subsequent BM degradation(32-48 hpf). In the current study we relied on pharmacological inhibition of VEGF signaling and mmp2 activity. Pharmacological inhibition can have potential off target effects, in particular developmental delays or toxicity. While we have partially controlled for these potential off target effects our future plans are to extend the analysis of these pathways with the use of genetic tools as to minimize potential for off target effects.

## Materials and Methods

### Zebrafish and embryo maintenance

Zebrafish were maintained using husbandry procedures approved by University of Kentucky IACUC committee. Embryos were kept at 28.5 °C in E3 embryo media. AB and TL strains were used as wild-type, Tg[*kdrl*:mCherry] transgenic line was used to visualize retinal vascularization^60^, Tg[*rx3*:GFP] was used to visualize retinal cells^61^.

Pax2^n^°^i^ embryos were a gift from Dr. Gregory-Evans. Genotyping analysis was conducted by amplifying the region of gDNA with the noi mutation using the forward primer: 5′-CTCGCTCTGCCTCCATGATTG3-′and the reverse: 5′-GGCACTGAAAGAGCACAGG-3′. The resultant 460 bp amplicon was digested with TaqI (NEB) which would recognize and digest the WT allele sequence but not the mutant allele.

Cloche (clo^m39^) mutant line was a gift from Dr. Mason Posner. Embryos were phenotyped for heart edema and genotyped as described by Reischauer *et al*., 2016^62^.

In all experiments, WT refers to unrelated wild-type embryos.

### Immunohistochemistry (IHC) and confocal imaging

Dechorionated embryos were fixed with 4% PFA in PBS at room temperature for 3 h and washed with PBST 4 times for 5 minutes. Embryos were then permeabilized with Proteinase K, 30 μg/mL 10 minutes for 24–28 hpf, 50 μg/mL 15-20 minutes for 32-48 hpf and 75 μg/mL 20 minutes for 56-72 hpf, washed 2 times in PBST for 5 minutes and blocked overnight at 4 °C with 10% sheep serum, 0.8% Triton X-100 and 1% BSA in PBS. Primary mouse anti-laminin antibody (ThermoFisher – 1:100) in blocking buffer (1% sheep serum, 1% BSA and 0.8% Triton X-100 in PBS) were incubated overnight at 4 °C and washed 5 times in PBST for 15 minutes. Secondary antibody, goat anti-rabbit (Alexa Fluor 488 – Abcam – 1:1000), DAPI 1:1000, and phalloidin (Alexa Fluor 555–1:50) were incubated overnight at 4 °C in the dark. Tg[*kdrl*:mCherry] embryos were treated with proteinase K as described above and stained with DAPI 1:1000 overnight. The embryos were washed 2 times in PBST for 15 minutes and visualized using a Nikon C2 + confocal microscope equipped with a 40×(1.15NA) water immersion objective. Embryos were embedded in 1.2% low melting point agarose on glass bottom 35 mm dishes (Fluorodish, World Precision Instruments). Images were captured in steps of 3.5 microns using Nikon Elements software. Image adjustment, such as cropping and brightness/contrast was performed using Adobe Photoshop. Images depicted for laminin and phalloidin staining are maximum projections of 3 planes (10.5 μm total) (Figs. 1,2,5, S4). Images of Tg[*kdrl*:mCherry] embryos are image snapshots of 3D reconstructions from entire z-stacks (Figs. 4–6). Images of fluorescent WISH are confocal single planes (Fig. 5).

### Analysis of fluorescence signal

Fiji software (https://fiji.sc) was used to measure the fluorescence intensity of laminin and phalloidin signal from raw image data. In order to account for variability in staining, normalization values were measured for laminin and actin pixel intensity where an area directly outside of the OF was measured and a ratio was generated between the two values (Fig. S1B). In cases where the fissure edges were farther apart then the size of the box used for analysis, the box width was divided into two and used to measure fluorescence intensity on each side of the fissure. Measured values from both boxes were averaged and then normalized. For Tg[*kdrl*:mCherry], 3D reconstructions of the OF were generated and individual cells were counted (from the opening of the OF through the back of the lens).

### Stats

Student’s t-test was used to compare individual time points. One-way ANOVA was used to analyze across treatments. Graphs are displayed as mean +/− standard deviation. Analysis was performed using Prism8 graphing software (GraphPad). ^*^p < 0.05, **p < 0.001, ***p < 0.0001.

### Whole mount *in situ* hybridization (WISH)

Whole mount *in situ* hybridization was performed as previously described^63^. RNA probes were generated using PCR with T7 promoter sequence linkers and subsequently transcribed [DIG or FITC labeled] using T7 polymerase (Roche). Primer sequences are all found in Table 1. Images were captured using a Nikon Digital sight DS-Fi2 camera mounted on a Nikon SZM800 stereo scope using Elements software. Dissected eyes from 24, to 72 hpf embryos were mounted in 70% glycerol and imaged under DIC using a Nikon TiE compound microscope equipped with a 20×(0.7NA) objective and Elements software. Image adjustment, such as cropping and brightness/contrast was performed using Adobe Photoshop.

**Table 1:** WISH and qPCR primer sequences.

WISH		
Gene	Prime Sequences	
Pax2a Probe FORWARD	ATGGATATTCACTGCAAAGCAG	
Pax2a Probe REVERSE	TAATACGACTCACTATAGGGCTAGTGGCGGTCATAGGCAGTG	
tln1 probe FORWARD	GCTATCGCTGTCACCGTTCAG	
tln1 T7 probe REVERSE	TAATACGACTCACTATAGGGGATGCCTTTGGTCATGCGGATG	
mmp2 probe FORWARD	ATGATGTCCGTTAAGTTTTTC	
mmp2 probe T7 REVERSE	TAATACGACTCACTATAGGGCTCAGGACAGAATCCGTAAGT	
mmp14a probe T7 REVERSE	TAATACGACTCACTATAGGGCACTCGCCAGAACCACTTTTC	
mmp14b probe FORWARD	ATGATGCTGATGTTTTGGACT	
mmp14b FORWARD	ATGATCTGGAGCGGGTTTACG	
mmp14b probe T7 REVERSE	TAATACGACTCACTATAGGGGAACCACTTACCCTTAAACAC	
vegfAA probe F	GAAGTTAATTTTAGCGGATTCG	
vegfAA probe R	TAATACGACTCACTATAGGGGTGATAGCAGCGACACACATTG	
vegfAB probe F	GCATGGGATTACTTCTGTGATG	
vegfAB probe R	TAATACGACTCACTATAGGGCTTCATGTCCGTTCTCAAGTC	
vegfCprobe F	ATGCACTTATTTGGATTTTCTG	
vegfC probe R	TAATACGACTCACTATAGGGCACACATTTGGTGCGGTTGAG	
rorb probe F		
rorb T7 probe R	TAATACGACTCACTATAGGGTCATTTGGGCATGACGGCGGC	
kdrl probe FORWARD	CAAGTGGCTAAAGGCATGGAG	
kdrl T7 probe REVERSE	TAATACGACTCACTATAGGGGACGGGTGGTGTGGAGTAACG	
**qPCR**		
**Gene**	**primer sequences**	
tln1	TTGGGGCAATTCCAGCAAAC	TGCTTCGCACAGGTTGTTAG
cldn5b	TACATCGTCGCAGGCTTGTT	GATGCTGCCCATCCGATGTA
tjp1a	CACAGAAGCAAGAGCCTGGA	AAAACCCGGTGCCCTGTG
flt4	CAGTCCAAAACAGCCAGCAC	CAACTCCACAGGCGAGTCTT
vegfaa	CAACGCGTATCGCAGCATAAT	AAGGCTCACAGTGGTTTTCTT
vegfab	TGTTGTATGTGACGGTGGGG	GCAAAACCGTGGTTCCAGAC
vegfc	TGGAGAAAGACGCTGTGCAT	TGCACTGAAGCTCCTCACTG
mmp2	TCAGGGTCGAGATGATGGGT	GAAAAGGAGCTCATGGGGACA
mmp14a	ACGCAGCTTATGAACGCAAT	TCCTGTGCCAAGCTCCTTAA
mmp14b	TCTTCAAAGGGGACAGGCAT	CACCAGTTCCCAGTTCTCCT

### qPCR analysis

32 hpf embryos were anesthetized with tricaine, tail tips were collected for genotyping and the heads, dissected just posterior of the eyes, were fixed in RNAlater. After genotyping, heads of embryos corresponding to WT and pax2a^−/−^ were pooled, 5-10 embryos, and total RNA isolated using a RNAaqueous kit (Ambion). DMSO and DMH4 treated embryos were harvested in the same fashion absent any genotyping. qPCR was performed as previously described^64^.

### Two color fluorescent *in situ* hybridization (FWISH)

Fluorescent whole mount *in situ* hybridization was performed as previously described^65^. Images were collected using a Nikon C2 + confocal microscope with a 20×(0.95NA) objective and images were adjusted for brightness and contrast using Adobe Photoshop.

### Live imaging analysis

Live imaging of Tg[kdrl:mCherry] embryos was conducted using a Nikon C2 + confocal microscope equipped with at 20×(0.95NA) water immersion objective. 22 hpf embryos were imbedded in 1.1% low gelling agarose in 1-inch glass bottomed Flourodish cell culture dishes (World Precision Instruments) and covered in embryo media, 3-amino benzoic acidethylester (tricaine) to anaesthetize the embryos and 1-phenyl 2-thiourea (PTU) to inhibit pigmentation. Z-stacks 75 μm thick with a step size of 2.5 μm were captured over the course of 6 hours at 10 minute intervals. The time lapse data were reconstructed in 3D using Elements software. Image adjustment, such as cropping and brightness/contrast was performed using Adobe Photoshop. After imaging, embryos were removed and genotyped.

### Inhibitor treatments

Embryos were incubated in embryo media with 5, 25, 50, or 100 $\mu \,$M of DMH-4 (Sigma Life Science) in DMSO starting at 12 hpf, unless otherwise stated. Fresh DMH4 containing media was added every 12 hpf for timepoints past 24 hpf. For ARP101 (Tocris) treatments embryos were dechorionated and treated with 10, 15, 20, 25 or 30 μM ARP101 in fish water from 24 to 48 hpf, unless stated otherwise. All experiments included 3+ biological replicates from separate clutches of embryos.

### Alt-R-crRNA CRISPR injections

Alt-R mmp2 crRNA, tracrRNA was pre-designed and synthesized by IDT. Duplex formation and dilution with Alt-R Cas9 v.3 enzyme was carried out as described by Hoshijima *et al*. 2019^44^.

### Ethics statement

The use of zebrafish in this study was approved by the University of Kentucky IACUC committee, Institutional PHS Assurance #D16-00217 (A3336-01) with a protocol number: 2015-1370. All experimental protocols were approved by the University of Kentucky Institutional Biosafety Committee, registration number B18-3186-M.

## Supplementary information

Supplementary information.

Supplementary information 2.

Supplementary information 3.

Supplementary information 4.

Supplementary information 5.

Supplementary information 6.

## Data Availability

All data and reagents generated in this manuscript will be freely available to any reasonable request. Sequences of primers used for probe synthesis are found in Table 1.
