# Supplementary material for: Hyaloid vasculature and mmp2 activity play a role during optic fissure fusion in zebrafish
Source: Sci Rep. 2020 Jun 23;10:10136. doi: 10.1038/s41598-020-66451-6 (PMC7311462; doi:10.1038/s41598-020-66451-6)

Hyaloid vasculature and mmp2 activity play a role during optic fissure fusion in zebrafish.

Megan L. Weaver[1], Warlen P. Piedade[1], Nishita N. Meshram, [1] and Jakub K. Famulski[1*]

1. Department of Biology, University of Kentucky

Supplemental Material

Figure S1: **Optic Fissure quantification**

A) Graphical representation of the region of OF analyzed using confocal microscopy. **B**) Sample image depicting regions of the OF (1), laminin normalization (2) and F-actin normalization (3) used for signal intensity quantification**. C)** Quantification of laminin and F-actin signal intensity in wild type embryos within the distal, medial and proximal regions of the OF, normalized to regions of laminin staining juxtaposed to the lens and F-actin signal within the lobe of the retina. Relative pixel intensities are displayed. ANOVA $p<0.0001$. **D)** Quantification of laminin and F-actin signal intensity in pax2a$^{-/-}$ embryos within the distal, medial and proximal regions of the OF, normalized to regions of laminin staining juxtaposed to the lens and F-actin signal within the lobe of the retina. Relative pixel intensities are displayed. ANOVA $p<0.0001$.

Figure S2: **Optic Fissure apposition measurements.**

Measurements of the distance between retinal lobes (apposition) in WT, pax2a$^{-/-}$ or DMH4 treated or embryos at 48hpf. Measurements were made using laminin staining as reference for edges of retinal lobes. Distance was measured as pixels.

Figure S3: ***pax2a* and *tln1* expression during development**

A) Whole mount in situ hybridization of *pax2a* probe at 24, 48, 54 and 72hpf. Lateral (top) and ventral (bottom) images depicting OF expression are shown. Pax2a expression persists in the fissure up to 54hpf. **B**) Whole mount in situ hybridization of *tln1* probe at 24, 48, 54 and 72hpf. Lateral (top) and ventral (bottom) images depicting OF expression are shown. *tln1* expression is detected in the OF from 24-48hpf (yellow arrowheads).

Figure S4: **DMH4 dose response**

A) 3D reconstructions of 48hpf whole mount Tg[*kdrl*:mCherry] (red) embryos treated with 1, 5, 50 or 100uM DMH4. DNA stained with DAPI (blue). Increasing concentration of DMH4 eliminates mCherry expressing cells from the OF and retina. Scale bar = 50μm. **B)** Brightfield images of DMSO or DMH4 treated embryos at 24, 48 and 72hpf. **C)** 3D reconstructions of whole mount Tg[*kdrl*:mCherry] (red) embryos treated with DMSO or 100μM DMH4 from 12-

24, 32, 36, 48 or 56hpf. DNA was stained with DAPI (blue). Scale bar = 50µm. **D)** Whole mount Immunohistochemistry was used to simultaneously visualize F-actin (red) and laminin (green) in DMSO treated embryos from 24-72hpf. Central-proximal regions of the OF are displayed. Scale bar = 50µm. **E)** Whole mount Immunohistochemistry was used to visualize laminin (green) and DNA (blue) 25 or 50µM DMH4 12-72hpf treatment. Central-proximal regions of the OF are displayed. Scale bar = 50µm.

Figure S5: **Time course of *mmp2, mmp14a* and *mmp14b* ocular expression**

A) Whole mount in situ hybridization of *mmp2, mmp14a, mmp14b* probes at 24, 26, 30 32, 34, 36 and 38hpf. *Mmp2* expression is present in the OF from 26-34hpf (yellow arrowheads). *Mmp14a* expression is present in the OF from 32-36hpf (yellow arrowheads). *Mmp14b* expression is present in the OF from 32-36hpf (yellow arrowheads). **B)** Whole mount in situ hybridization comparing *mmp2, mmp14a* and *mmp14b* expression in WT vs pax2a$^{-/-}$ and DMSO vs DMH4 treated embryos at 32hpf. *Mmp2, 14a and 14b* signal within the OF (yellow arrowhead) appears decreased in pax2a$^{-/-}$ and DMH4 treated embryos.

Figure S6: **ARP101 treatment dose response**

A) Brightfield images of DMSO and ARP treated embryos at 48hpf. Concentrations of ARP101 exceeding 20uM result in toxic effects. **B)** Whole mount Immunohistochemistry was used to visualize laminin (red) in ARP101 treated Tg[*rx3*:GFP] embryos at 48hpf. Central-proximal and distal regions of the OF are displayed. Scale bar = 50µm. **C)** Fluorescent whole mount in situ hybridization of *pax2a* probe at 32hpf in DMSO or 20µM ARP101 treated embryos. OF expression is indicated with a yellow arrowhead. ARP101 treatment does not appear to alter *pax2a* expression in the OF. Broken white lines outline the retinal lobes. Scale bar = 50µm.

Figure S7: **mmp2 Alt-R crRNA injection results in OF fusion failure phenotypes.**

Brightfield images of **A)** uninjected, **B)** *mmp2* crRNA or **C)** pax2a$^{-/-}$ embryos at 72hpf. OF fusion failure is observed as a notch in the ventral retina in both *mmp2* crRNA injected embryos as well as in pax2$^{-/-}$. Confocal stacks of laminin stained embryos at 72hpf from **D)** uninjected, **E)** *mmp2* crRNA and **F)** pax2a$^{-/-}$ embryos. Pax2a$^{-/-}$ embryos display a clear fissure at 72hpf, while *mmp2* crRNA injected embryos achieve only partial fusion when compared to uninjected

controls. **G)** *mmp2* crRNA efficacy was assayed using PCR flanking the region targeted. Lanes 1-8 represent gDNA assays from individual embryos assayed at 72hpf. Control is found in well 9. ** indicate changes in band size compared to control. $ corresponds to embryo imaged in B and E.

Figure S8: **mmp2 expression in *cloche* mutants**
Whole mount in situ hybridization of *mmp2* expression at 32hpf in $clo^{+/+}$ and $clo^{m39/m39}$ embryos. Individual dissected eyes were imaged at 20X using DIC optics. Yellow arrowheads indicate OF expression in wholemount samples. Magenta arrowheads indicate expression within the OF as observed at higher magnification in dissected eyes.

Movie 1: WT Tg[*kdrl*:mCherry] migration within the OF, 24-30hpf

Movie 2: $pax2a^{-/-}$ Tg[*kdrl*:mCherry] migration within the OF, 24-30hpf

Movie 3: 3D rotation of WT Tg[*kdrl*:mCherry] embryo at 48hpf

Movie 4: 3D rotation of $pax2a^{-/-}$ Tg[*kdrl*:mCherry] embryo at 48hpf

Movie 5: 3D rotation of ARP101 treated Tg[*kdrl*:mCherry] embryo at 48hpf

Supplementary Figure 1

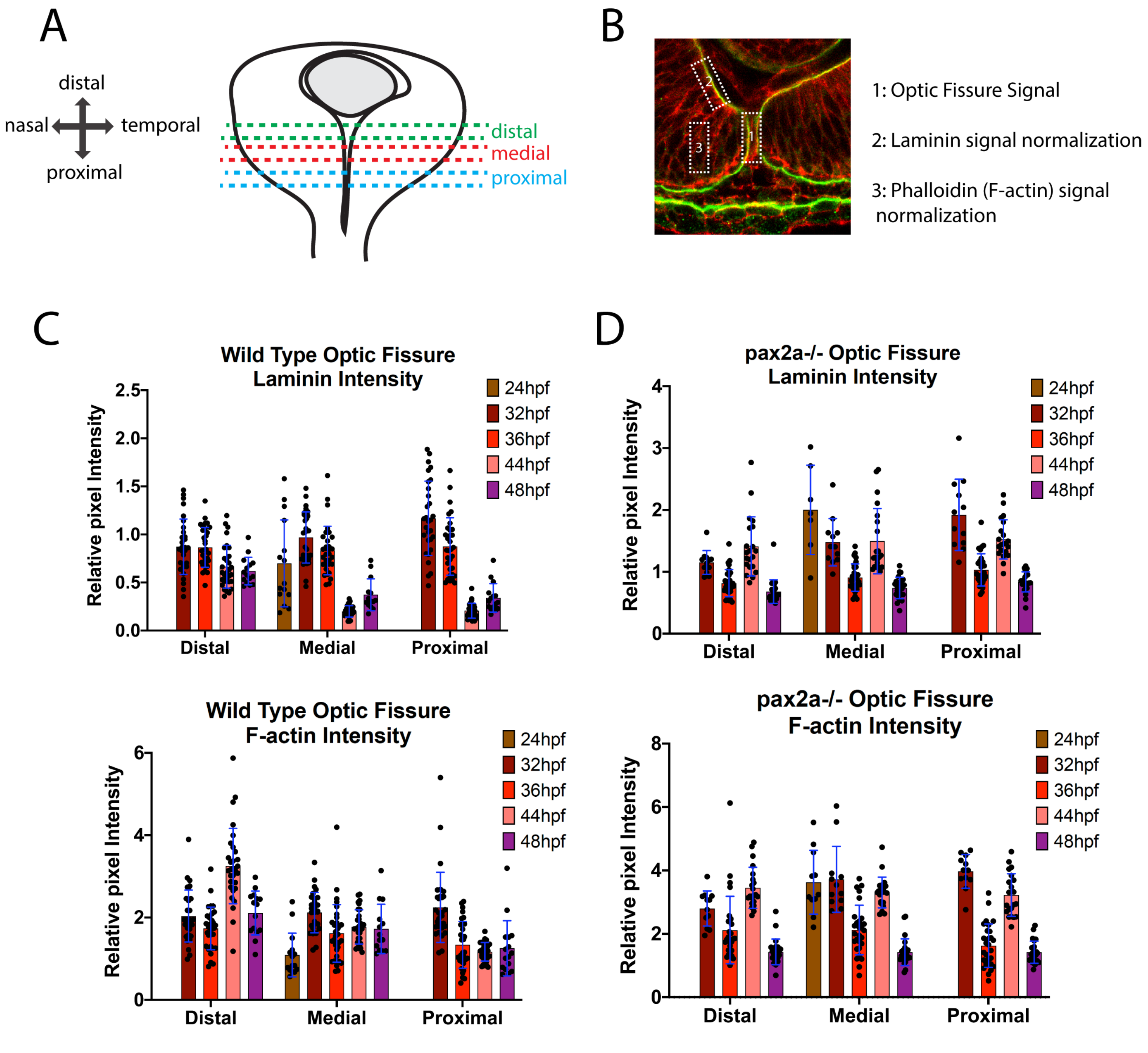

Supplementary figure 2

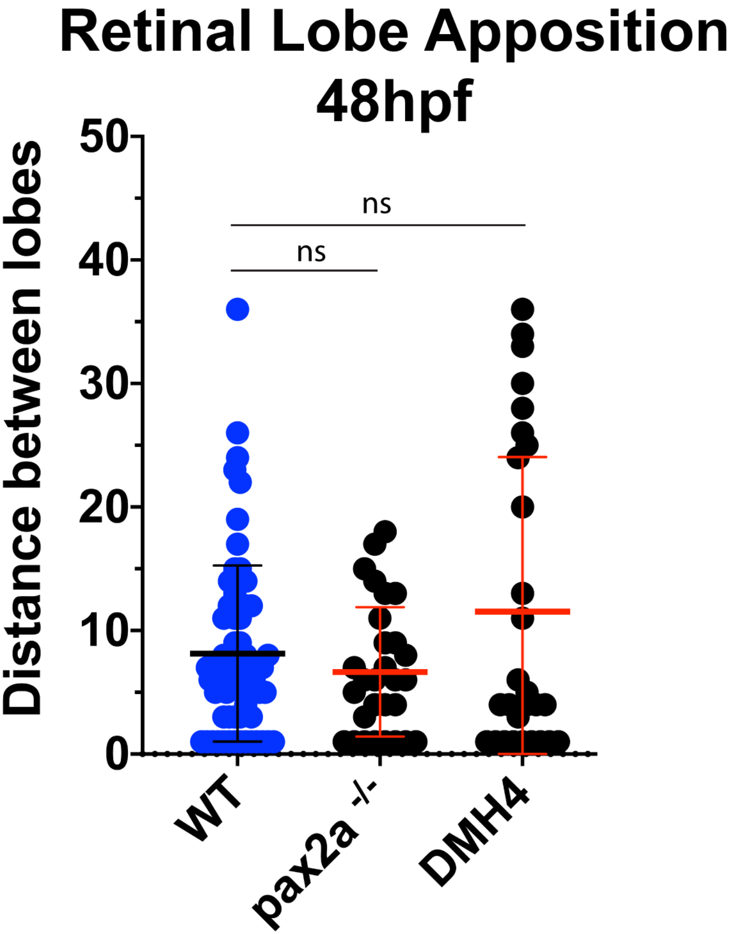

Supplementary Figure 3

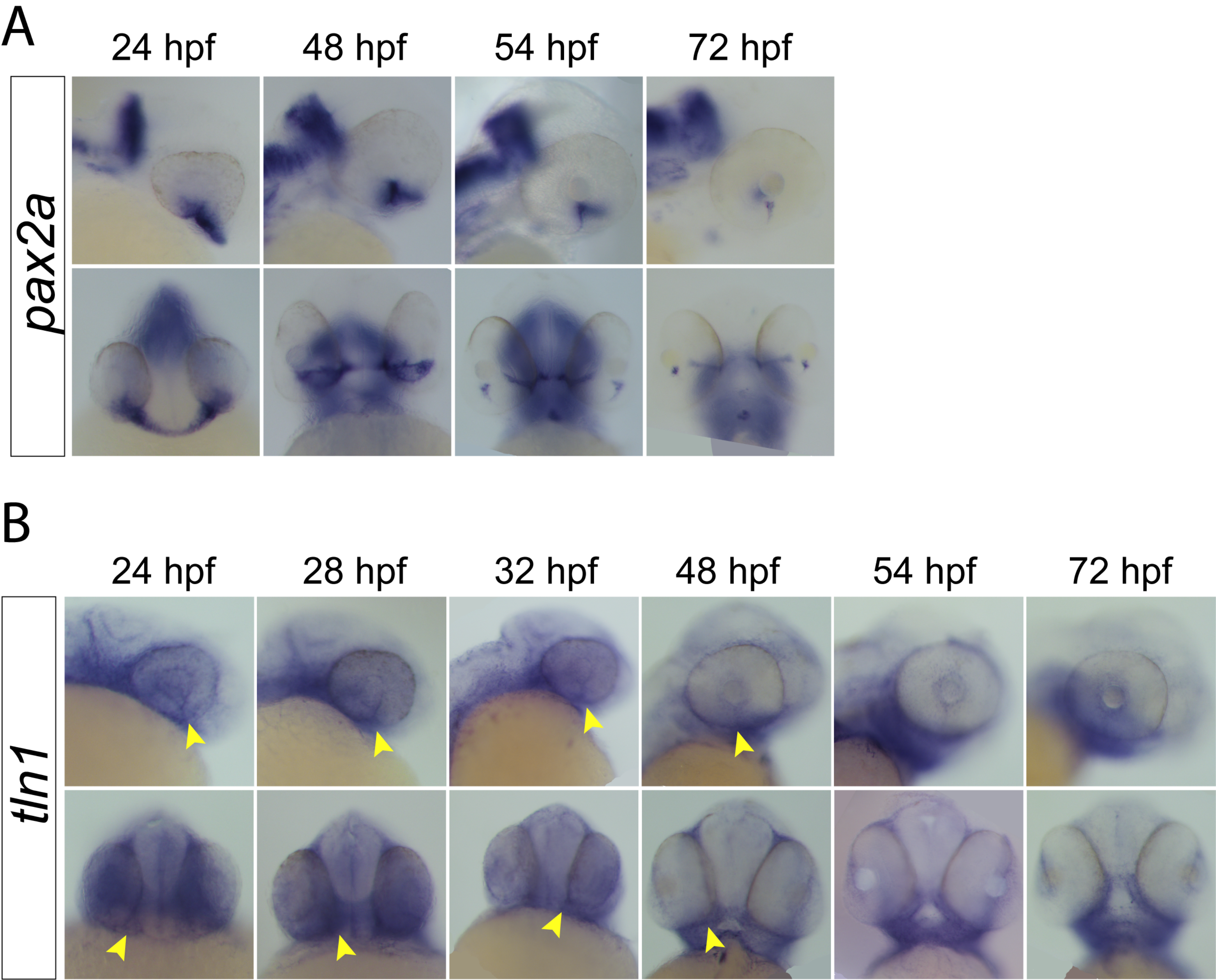

Supplementary Figure 4

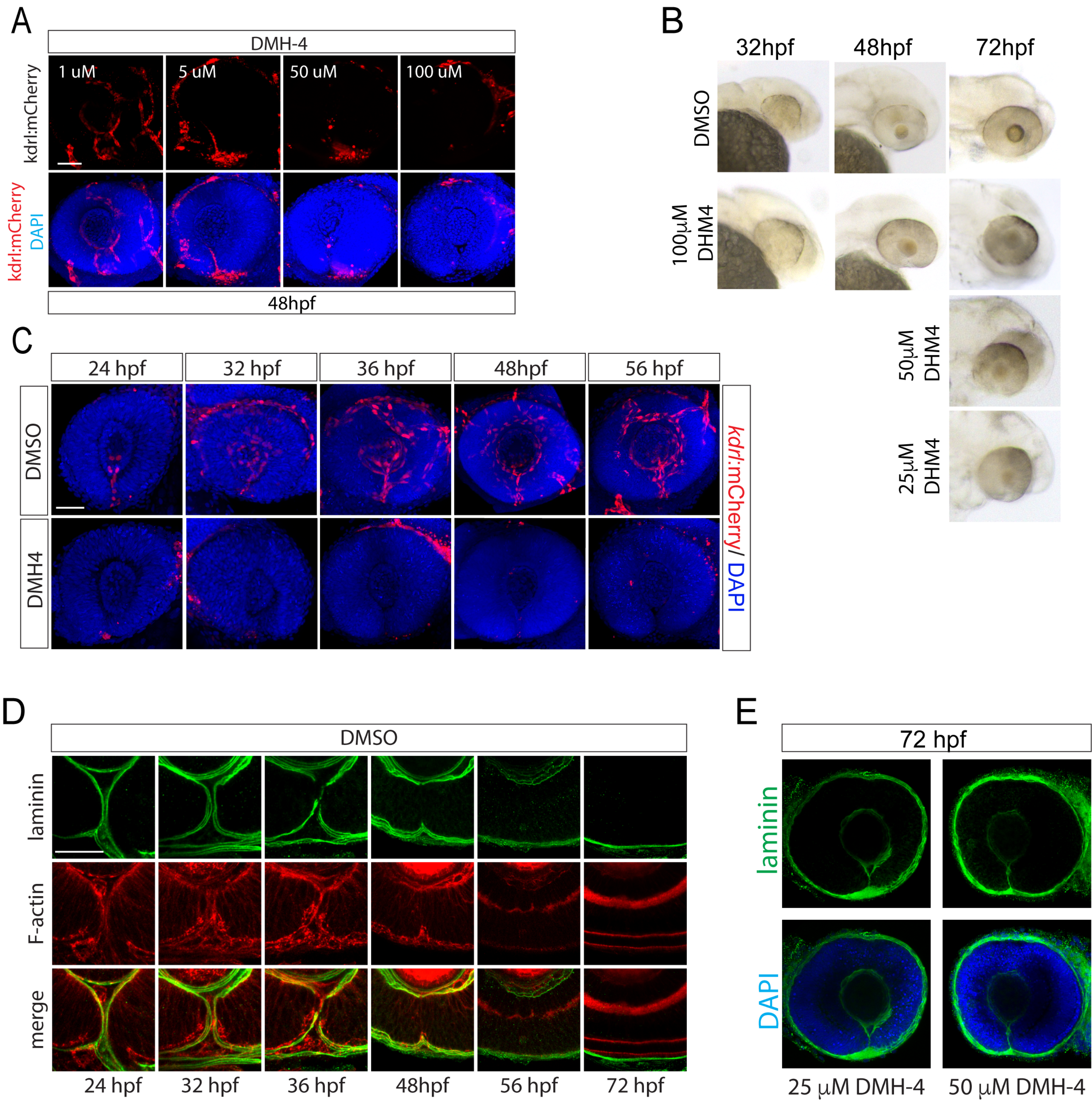

Supplementary Figure 5

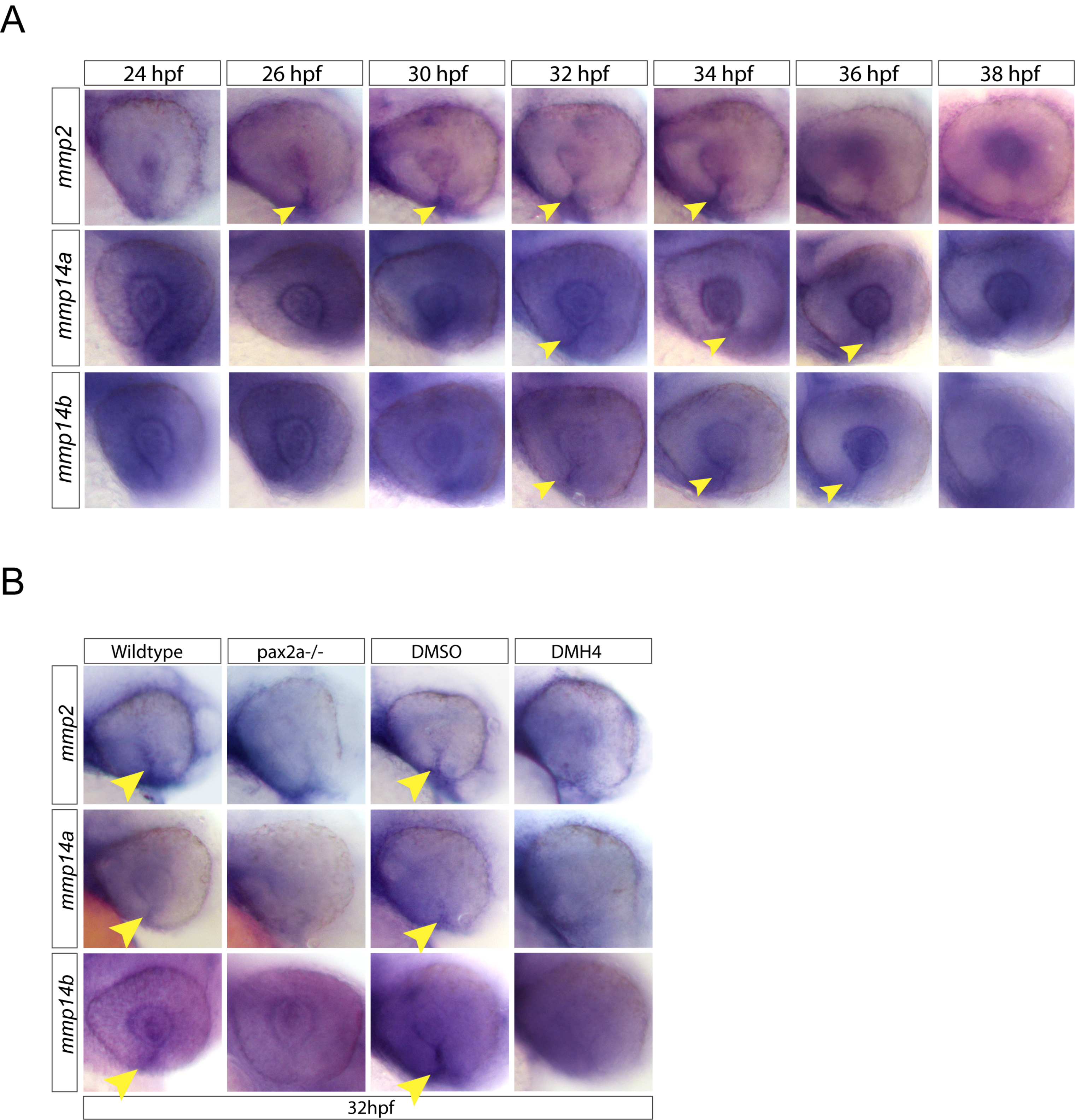

Supplementary Figure 6

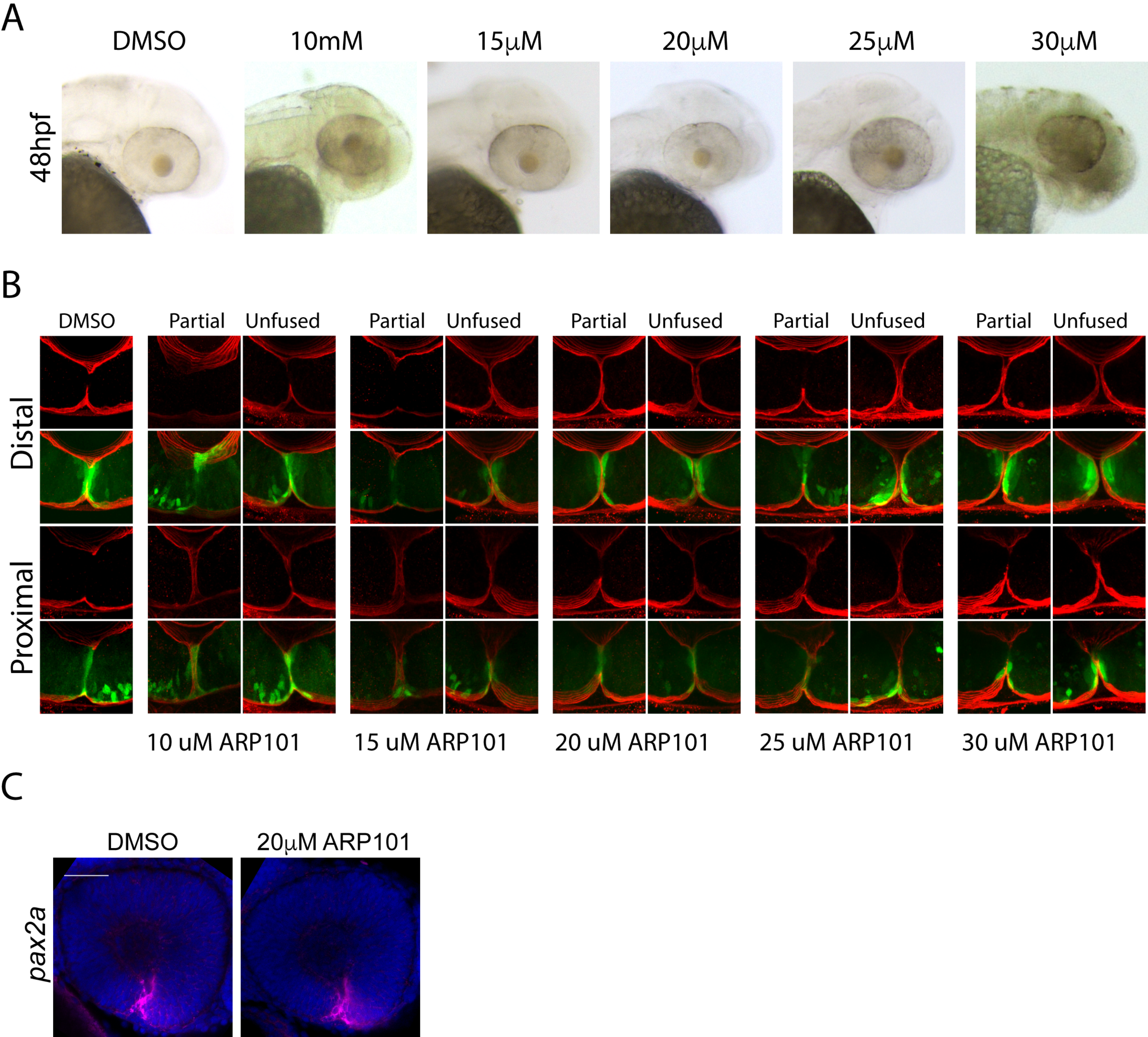

Supplemental Figure 7

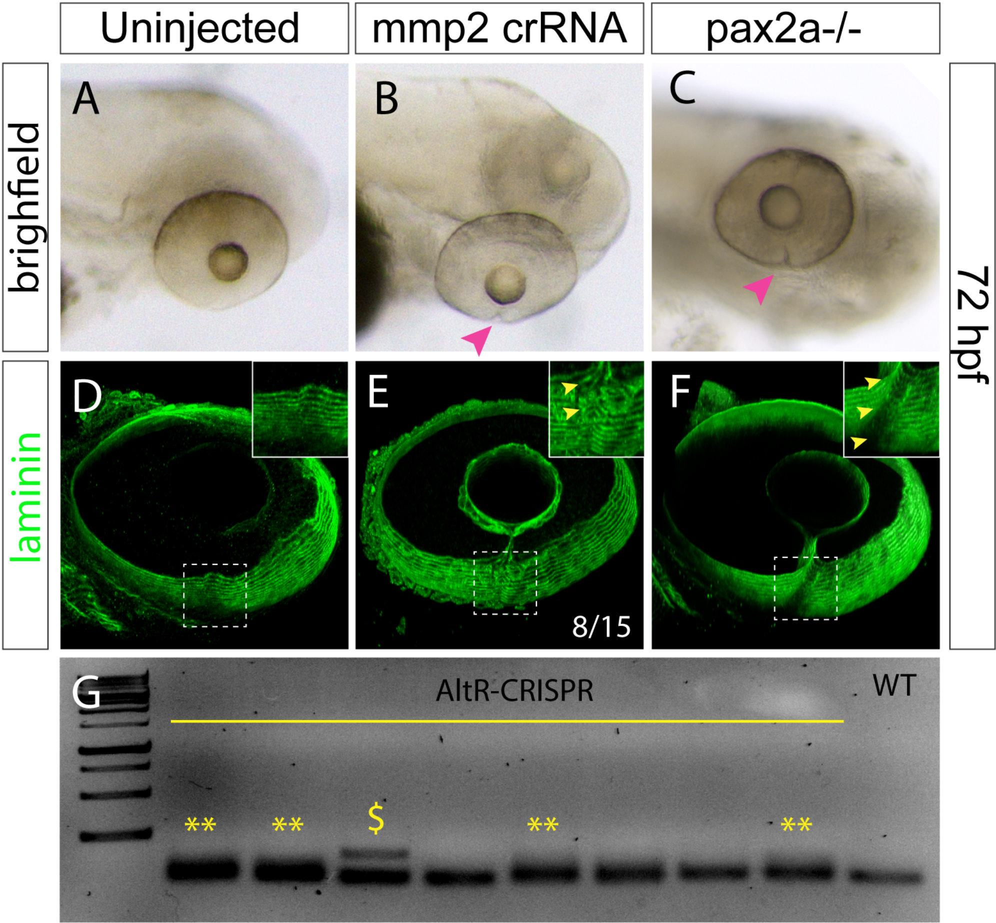

Supplemental Figure 8

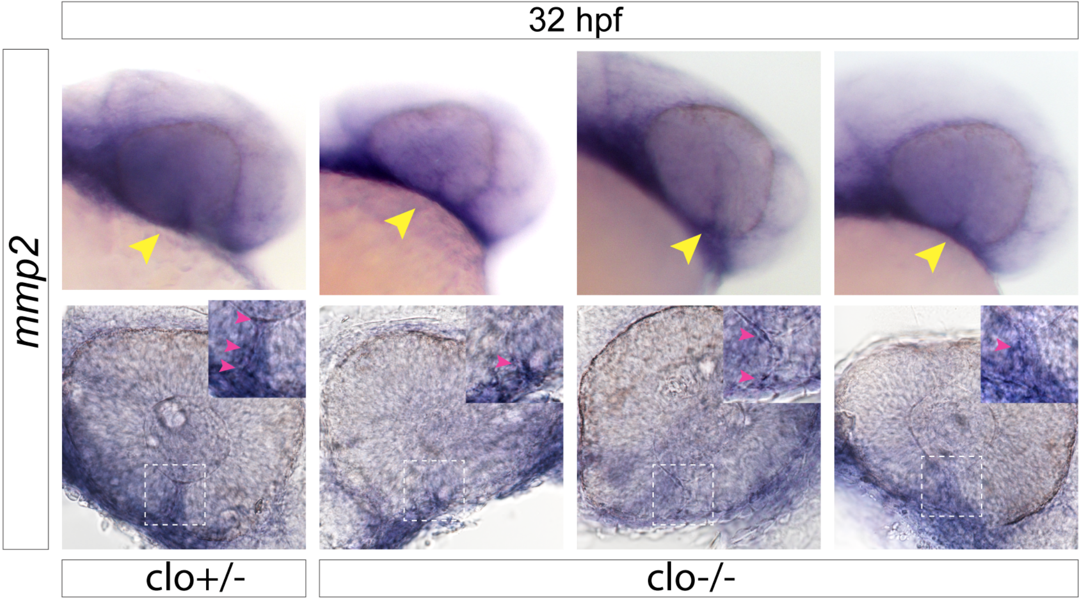

Supplement: Supplementary file 6 — Supplementary information 6. [file 41598_2020_66451_MOESM6_ESM.pdf]
